# Recent Advances on Sodium‐Ion Batteries and Sodium Dual‐Ion Batteries: State‐of‐the‐Art Na^+^ Host Anode Materials

**DOI:** 10.1002/smsc.202100014

**Published:** 2021-05-05

**Authors:** Decai Gong, Chenyang Wei, Zhongwang Liang, Yongbing Tang

**Affiliations:** ^1^ Functional Thin Films Research Center Shenzhen Institute of Advanced Technology Chinese Academy of Sciences Shenzhen 518055 China; ^2^ Nano Science and Technology Institute University of Science and Technology of China Suzhou 215123 China; ^3^ School of Chemical Sciences University of Chinese Academy of Sciences Beijing 100049 China; ^4^ Key Laboratory of Advanced Materials Processing and Mold Ministry of Education Zhengzhou University Zhengzhou 450002 China

**Keywords:** Na^+^ host anode materials, sodium dual-ion batteries, sodium storage mechanisms, sodium-ion batteries

## Abstract

Sodium is abundant on Earth and has similar chemical properties to lithium, thus sodium‐ion batteries (SIBs) have been considered as one of the most promising alternative energy storage systems to lithium‐ion batteries (LIBs). Meanwhile, a new energy storage device called sodium dual‐ion batteries (SDIBs) is attracting much attention due to its high voltage platform, low production cost, and environmental benignity coming from the feature of directly using graphite as the cathode. However, due to the large mass and ionic radius of sodium atoms, SIBs and SDIBs exhibit low energy density and inferior cycling life than LIBs. Over the past few years, tremendous efforts, especially in the area of anode materials, have been made to improve the electrochemical performance of SIBs and SDIBs. Reviewing and summarizing the previous studies will be helpful for future exploration and optimization. Herein, the recent progress on anode materials for SIBs and SDIBs is summarized according to the reaction mechanism. The structural design, reaction mechanism, and electrochemical performance of the anode materials are briefly discussed. In addition, the fundamental challenges, potential solutions, and perspectives in this field are also proposed. It is hoped that this Review may advance the development of anode materials for sodium storage.

## Graphical abstract

The recent progress on anode materials for both sodium‐ion batteries and sodium dual‐ion batteries are summarized according to the different sodium storage mechanisms. The structural design, reaction mechanism, and electrochemical performance of the anode materials are briefly discussed. In addition, the fundamental challenges, potential solutions, and perspectives in this field are also presented.

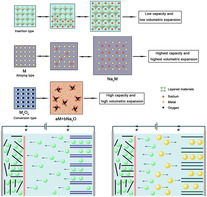

## 1 Introduction

Nowadays, the main energy sources for human consumption are still traditional fossil fuels, which have provided the vast majority of world energy consumption.^[^ ^1^ ^]^ Nevertheless, fossil fuels are nonrenewable energy sources and will run out one day. In addition, the extensive depletion of fossil fuels will cause the emission of massive greenhouse gas (carbon dioxide), which would sharply accelerate the trend of global warming. Thus, exploiting clean and renewable energy sources such as wind, solar, hydro, and tidal energy is extremely important for the long‐term development of human beings.^[^ ^2^ ^]^ However, due to their intermittency and unbalanced geographical distribution nature, these renewable energy sources cannot be used directly. It is necessary to develop efficient and low‐cost energy storage and conversion techniques to make use of them. Due to the high energy densities and flexibility, rechargeable batteries are the most widely used energy storage device at present.^[^ ^3^ ^]^ Among them, lithium‐ion batteries (LIBs) have the most mature technology and extensive commercial applications, which have captured the main market of electric vehicles, portable electronic devices, and large‐scale stationary energy storage.^[^ ^4^ ^]^ However, lithium is actually not abundant in the Earth's crust, and it is estimated that a quarter of lithium reserves are expected to be depleted by 2025.^[^ ^5^ ^]^ As a result, the cost of producing LIBs is set to rise further in the future, which will immensely restrict their large‐scale applications. Therefore, developing alternative battery technology with low cost and outstanding performance is under urgent demand.^[^ ^6^ ^]^

In recent years, Na^+^ batteries, including sodium‐ion batteries (SIBs) and sodium dual‐ion batteries (SDIBs), have been extensively investigated due to the low cost, sustainability, and natural abundance of sodium resources.^[^ ^7^ ^]^ In addition, because of less toxicity of sodium salts (NaPF_6_, NaFSI, and NaTFSI) compared with lithium salts (LiPF_6_, LiFSI, and LiTFSI), sodium‐based electrolytes are more environmentally friendly than lithium‐based electrolytes.^[^ ^8^ ^]^ All these advantages make Na^+^ batteries suitable for large‐scale energy storage systems with low cost, environmental friendliness, and high performance in the future. Up to now, massive efforts have been made to apply the ripe experience on LIBs to Na^+^ batteries, especially in developing appropriate Na‐host electrode materials with fast Na^+^ insertion/extraction, high specific capacity, and long cycling lifespan.^[^ ^9^ ^]^ However, Na^+^ has a larger ionic radius than Li^+^ (1.02 Å for Na^+^ vs 0.76 Å for Li^+^), which would cause sluggish reaction kinetics and structural instability of electrode materials during the charge/discharge process, leading to lower capacity, poor rate capability, and short cycling lifespan of the Na^+^ batteries.^[^ ^10^ ^]^ In addition, sodium atom is much heavier than lithium atom (23 g mol^−1^ for Na vs 6.9 g mol^−1^ for Li), and the redox potential of Na/Na^+^ couple (−2.71 V vs standard hydrogen electrode) is also higher than Li/Li^+^ couple (−3.02 V vs standard hydrogen electrode), which would further lead to a lower energy density of the Na^+^ batteries.^[^ ^11^ ^]^ Therefore, it is important to exploit satisfying electrode materials with low cost and superior performance for Na^+^ batteries.

**Figure 1:**
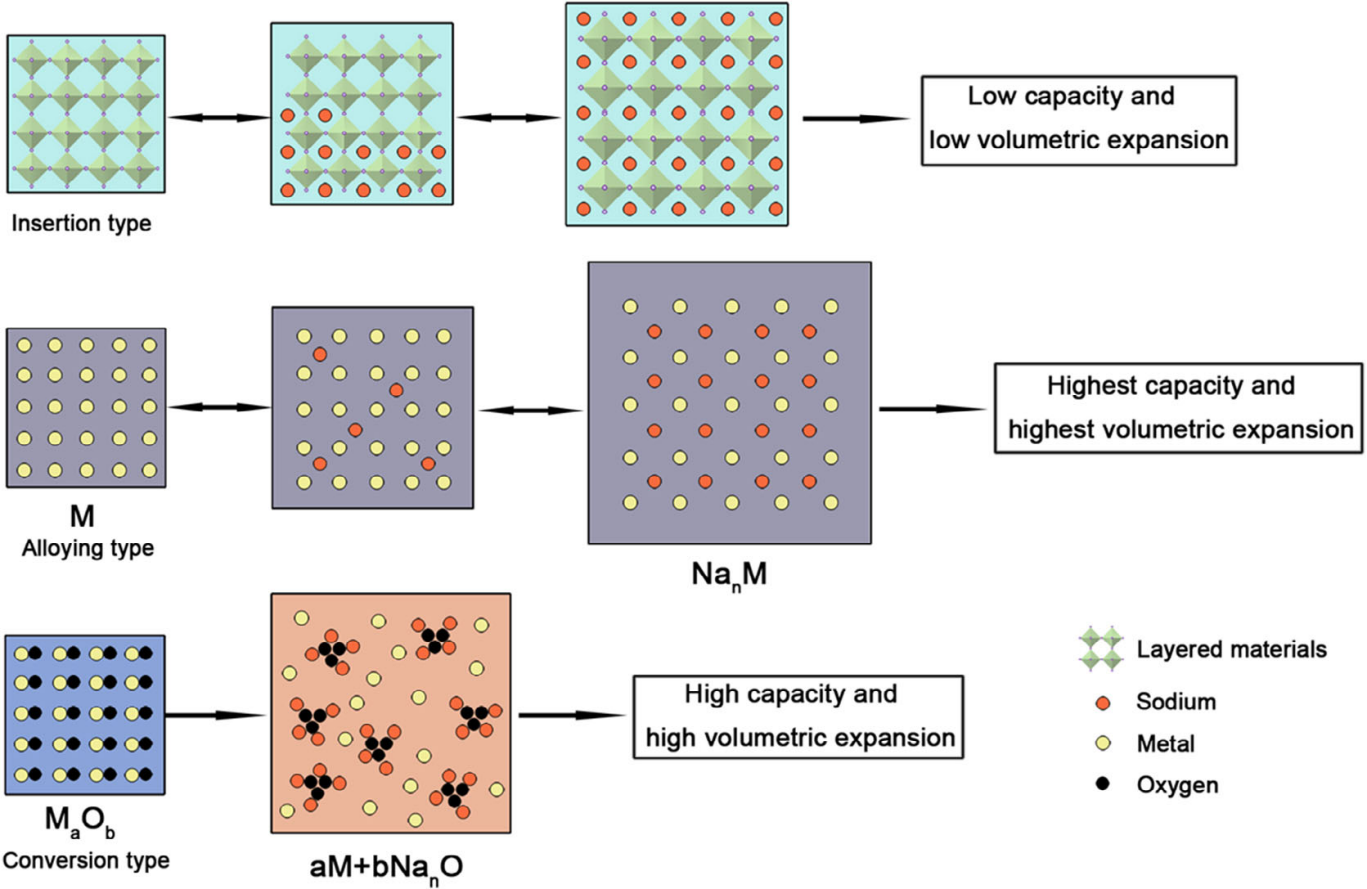
Schematic illustration of three types of reaction mechanisms for sodium storage in different kinds of anodes.

Sodium metal has a high theoretical capacity (1166 mAh g^−1^); however, the low melting point and formation of sodium dendrites would cause a severe safety hazard.^[^ ^12^ ^]^ As a result, researchers have to be driven to develop other desirable anode materials. Up to now, there are mainly three types of anode materials for Na^+^ storage based on the different reaction mechanisms: 1) insertion reaction mechanisms, 2) alloying reaction mechanisms, and 3) conversion reaction mechanisms (**Figure** **1**).^[^ ^13^ ^]^ So far, considerable efforts have been made to improve the electrochemical performance of these anode materials; however, each type of material still has some obvious drawbacks. For instance, as the most widely investigated insertion type anode materials, hard carbon usually exhibits low capacity and inferior rate capability.^[^ ^14^ ^]^ While the alloying‐ and conversion‐type materials that can provide capacity two or three times higher than the insertion type would suffer from a remarkable volumetric change during the sodiation process, which can cause the exfoliation of active material from the current collector, leading to poor cycling stability.^[^ ^15^ ^]^ As a result, seeking for satisfying anode materials is a great challenge for the development of advanced Na^+^ batteries. Up till now, there are some excellent reviews that summarize the anode materials for Na^+^ storage in SIBs, but a large number of frontier and important studies have been reported in recent years. For example, in the research on sodium storage mechanism, a new sodium storage mechanism, including a three‐step process for hard carbon, was proposed in recent years, which is quite different from the traditional two‐step card‐house model, indicating a more complex process on Na^+^ storage in hard carbon.^[^ ^14^, ^16^ ^]^ In addition, in situ characterizations techniques, such as in situ transmission electron microscopy (TEM), have been increasingly used to study the reaction between Na^+^ and anode materials, which could let us take a closer look at how Na^+^ are stored and further improve the properties of sodium storage.^[^ ^17^ ^]^ Most important of all, there is no specific review to focus on the anode materials for SDIBs. Although SDIB is a newly developing battery system that emerged in the past 5 years, it shows a good application prospect due to its high energy density and low cost.^[^ ^18^ ^]^ Therefore, it is necessary to review the recent progress on anode materials for SIBs and systematically summarize the anode materials for SDIBs.

In this Review, we begin with a brief introduction of the working principle of SIBs and SDIBs. Then, the most recent progress on anode materials for high‐performance SIBs, including typical insertion‐type materials (carbonaceous materials), alloying‐type materials (metal materials and phosphorus materials), and conversion‐type materials (metal oxides/phosphides/sulfides), is summarized in detailed. Next, we systematically summarize the appropriate anode materials for SDIBs reported in the past 5 years. At last, the current facing challenges are discussed, and some possible perspectives are also provided to further promote the development of SIBs and SDIBs.

## 2 Working Principle of SIBs and SDIBs

**Figure 2:**
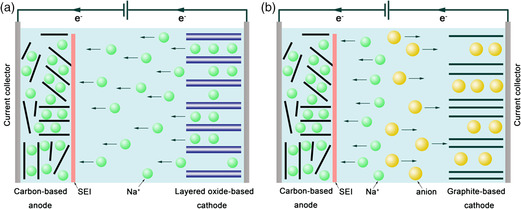
Schematic illustration of the working mechanisms of a) SIBs and b) SDIBs during the charging process.

With electrochemical mechanisms similar to LIBs, SIBs have been widely studied and regarded as the most promising alternative energy storage devices to LIBs in the future.^[^ ^19^ ^]^ The operation mechanism of SIBs can be described as the rocking‐chair model, in which only Na^+^ ions are shuttled between the anode and cathode. Normally, the shuttling Na^+^ is coming from the layered oxide‐based cathode. The electrolytes only serve to transfer Na^+^, which actually do not participate in the energy storage process, as shown in **Figure** **2**a. In addition, a new battery system called SDIBs is attracting more and more attention from researchers due to its high voltage platform, low production cost, as well as environmental benignity.^[^ ^18^ ^]^ It is known for decades that anions can insert into host materials such as graphite at high potential, which forms the theoretical basis of the current dual‐ion batteries (DIBs).^[^ ^20^ ^]^ In 2015, Bordet et al. pioneered the work of SDIBs by investigating the intercalation of anions into graphite in sodium‐based electrolytes.^[^ ^21^ ^]^ Two years later, our group reported a novel tin–graphite SDIB (tin as anode and graphite as the cathode) with a high energy density.^[18a]^ Afterward, Lu and coworkers further advanced the research of SDIBs using soft carbon as anode materials, indicating potential application prospects.^[^ ^11^, ^22^ ^]^ The operation mechanism of SDIBs is quite different from that of SIBs, in which both Na^+^ and anions in the electrolytes participate in the energy conversion process (Figure 2b). During the charging process, Na^+^ and anions in the electrolytes are stored into anode and cathode, respectively, whereas during the discharge process, both ions will be released into the electrolytes again.^[^ ^23^ ^]^ Actually, the operation mechanism of SDIBs is similar to that of supercapacitors, in which cations and anions are separated and stored into anode and cathode through capacitive behaviors, respectively.^[^ ^24^ ^]^ The major difference is that the storage of ions in SDIBs is based on battery‐type redox reactions such as insertion reactions in the cathode at high potentials, as well as insertion, alloying, or conversion reactions in the anode at low potentials, thus leading to high energy density.

## 3 Anodes for SIBs

The development of appropriate anode materials is the key to the large‐scale application of SIBs. As discussed earlier, Na metal is not suitable to be used as the anode for SIBs due to its safety hazards. Therefore, it is critical to exploit the authentic Na^+^ host materials, in which Na^+^ can cycle back and forth in a rocking‐chair form. Generally, the research of anodes for SIBs mainly focuses on the materials based on the mechanism of insertion, alloying, and conversion reactions.^[^ ^1^, ^25^ ^]^ As typical insertion anode materials, carbonaceous materials, especially hard carbon materials, have been widely studied due to their low cost and good cycling stability.^[^ ^14^, ^26^ ^]^ In addition, the metal and phosphorus materials driven by the alloying reaction, as well as metal oxides/phosphides/sulfides materials driven by conversion reaction, have attracted more and more attention as anode for SIBs due to their high specific capacity. However, the poor‐rate capability and low capacity of carbonaceous materials, and huge volumetric expansion of the alloying and conversion‐type materials dramatically hinder their further application.^[^ ^27^ ^]^ Moreover, compared with LIBs, the sluggish reaction kinetics of SIBs caused by the large size of Na^+^ is another issue that needs to be addressed.^[^ ^28^ ^]^ Therefore, more efforts should be made to modify electrode materials and improve their electrochemical performance. In this section, the recent progress in anode materials for SIBs is discussed and their electrochemical performances are shown in **Table** **1**.

**Table 1:** Summary of electrochemical performances of recent progress on anode materials for SIBs

No	Anode materialsa)	Reaction mechanism	Electrolytes	Cycling performance [mAh g^−1^]	Rate capability [mAh g^−1^]	References
1	Graphite	Insertion	1 m NaOTf in DME	≈90 at 37.2 mA g^−1^ after 1000 cycles	≈75 at 372 mA g	[33]
2	N‐rich mesoporous carbon	Insertion	1 m NaClO_4_ in EC/DEC (1:1, v/v)	252 at 50 mA g^−1^ after 100 cycles	49.8 at 2 A g	[103]
3	Ca‐rich hard carbon	Insertion	1 m NaClO_4_ in PC with 5% FEC	326.7 at 50 mA g^−1^ after 250 cycles	43.9 at 1 A g	[37]
4	N‐rich interpenetrated porous hard carbon	Insertion	1 m NaPF_6_ in DEGDME	250 at 1 A g^−1^ after 500 cycles	239.8 at 2 A g	[38]
5	Low‐defect and low‐porosity hard carbon	Insertion	1 m NaClO_4_ in EC/DEC (1:1, v/v)	340 at 20 mA g^−1^ after 100 cycles	/	[39]
6	Vertical graphene and N‐doped carbon	Insertion	1 m NaClO_4_ in EC/DMC (1:1, v/v) with 5% FEC	398 at 1 A g^−1^ after 1000 cycles	300 at 2 A g	[104]
7	3D amorphous carbon with controlled porous and disordered structures	Insertion	1 m NaPF_6_ in EC/DMC (1:1, v/v)	188 at 0.3 A g^−1^ after 600 cycles	66 at 9.6 A g	[105]
8	3D hard carbon matrix	Insertion	1 m NaClO_4_ in PC	116 at 4 A g^−1^ after 3000 cycles	72 at 10 A g	[106]
9	Porous carbon nanofibers	Insertion	1 m NaClO_4_ in EC/DEC (1:1, v/v)	266 at 50 mA g^−1^ after 100 cycles	40 at 20 A g	[42]
10	Hard carbon nanoparticles co‐doped with N, S	Insertion	1 m NaClO_4_ in EC/DEC (1:1, v/v) with 5% FEC	223 at 1 A g^−1^ after 2000 cycles	102 at 10 A g	[45]
11	Wood cellulose fibers derived hard carbon	Insertion	1 m NaClO_4_ in EC/DEC (1:1, v/v)	200 at 100 mA g^−1^ after 200 cycles	/	[43]
12	Soft carbon nanosheets	Insertion	1 m NaClO_4_ in EC/DMC/EMC (1:1:1, v/v/v)	128.7 at 800 mA g^−1^ after 3500 cycles	103.8 at 1 A g	[29]
13	Nanostructured Sn anchored on graphene sheets	Alloying	1 m NaClO_4_ in PC/FEC (9:1,v/v)	324 at 50 mA g^−1^ after 30 cycles	90 at 0.4 mA g	[107]
14	Pipe‐wire TiO_2_‐Sn@carbon nanofibers	Alloying	1 m NaClO_4_ in EC/DMC (6:4, v/v)	413 at 100 mA g^−1^ after 400 cycles	/	[57]
15	Sn@CNT nanopillars grown perpendicularly on carbon paper	Alloying	1 m NaClO_4_ in EC/PC (1:1, v/v) with 5% FEC	445 μAh/cm^2^ at 250 μA cm^−2^ after 100 cycles	299 μAh/cm^2^ at 1000 μA cm^−2^	[58]
16	Yolk‐shell Sn@C	Alloying	1 m NaClO_4_ in EC/DEC (1:1, v/v) with 1% FEC	≈200 at 1000 mA g^−1^ after 1000 cycles	≈200 at 5 A g	[108]
17	Sn nanodots embedded inside N‐doped carbon microcages	Alloying	1 m NaClO_4_ in PC with 5% FEC	332 at 500 mA g^−1^ after 300 cycles	≈200 at 5 A g	[109]
18	Sn nanodots encapsulated in porous N‐doped carbon nanofibers	Alloying	1 m NaClO_4_ in PC with 5% FEC	484 at 2 A g^−1^ after 1300 cycles	450 at 10 A g	[56]
19	Sb@C coaxial nanotubes	Alloying	1 m NaClO_4_ in PC with 5% FEC	407 at 0.1 A g^−1^ after 240 cycles	310 at 20 A g	[59]
20	Sb@C microspheres	Alloying	1 m NaPF_6_ in EC/DEC (1:1, v/v) with 5% FEC	584 at 200 mA g^−1^ after 100 cycles	302 at 3 A g	[110]
21	Sb@multilayer graphene hybrid	Alloying	1 m NaClO_4_ in EC/DEC/FEC (1:1:0.1, v/v/v)	406 at 100 mA g^−1^ after 200 cycles	210 at 5 A g	[111]
22	Yolk‐shell structured Sb@C	Alloying	1 m NaClO_4_ in EC/DMC (1:2, w/w) with 10% FEC	422 at 500 mA g^−1^ after 200 cycles	315 at 5 A g	[62]
23	Sb PHMSs	Alloying	1 m NaClO_4_ in PC with 5% FEC	617 at 100 mA g^−1^ after 100 cycles	312.9 at 3.2 A g	[63]
24	Spherical nano‐Sb@C composite	Alloying	1 m NaClO_4_ in PC with 5% FEC	350 at 100 mA g^−1^ after 500 cycles	≈270 at 4 A g	[112]
25	N‐doped carbon hollow nanotube encapsulated Sb nanorod composite	Alloying	1 m NaClO_4_ in EC/DMC (1:1, w/w) with 5% FEC	395 at 2 A g^−1^ after 3000 cycles	379.9 at 10 A g	[113]
26	Yolk‐Shell Bi@Void@C nanospheres	Alloying	1 m NaPF_6_ in DME	198 at 20 A g^−1^ after 10 000 cycles	173 at 100 A g	[114]
27	Bi@graphite	Alloying	1 m NaPF_6_ in DME	142 at 3.2 A g^−1^ after 10 000 cycles	≈110 at 48 A g	[115]
28	Bi nanoparticle@carbon	Alloying	1 m NaPF_6_ in DME	265 at 8 A g^−1^ after 30 000 cycles	232 at 60 A g	[116]
29	Bi nanoflakes on Ni foam	Alloying	1 m NaPF_6_ in DEGDME	302.4 at 200 mA g^−1^ after 100 cycles	206.4 at 2 A g	[117]
30	Multicore‐Shell Bi@N‐doped carbon nanospheres	Alloying	1 m NaPF_6_ in DME	235 at 10 A g^−1^ after 2000 cycles	211 at 50 A g	[118]
31	Hollow RP sphere	Alloying	1 m NaClO_4_ in EC/DMC (1:1, v/v) with 5% FEC	1500 at 1300 mA g^−1^ after 80 cycles	278 at 10.4 A g	[119]
32	RP@graphene with 70 wt% P	Alloying	1 m NaClO_4_ in EC/DEC (1:1, v/v) with 10% FEC	1700 at 520 mA g^−1^ after 60 cycles (for P)	520 at 5.2 A g^−1^ (for P)	[120]
33	Porous RP/RGO with 67.6 wt% P	Alloying	1 m NaClO_4_ in PC with 5% FEC	524 at 3465.3 mA g^−1^ after 1500 cycles	656.9 at 3465.3 mA g	[121]
34	RP@N‐doped carbon nanofibers with 51 wt% P	Alloying	1 m NaClO_4_ in PC with 5% FEC	619 at 2000 mA g^−1^ after 1000 cycles	343 at 10 A g	[122]
35	RP@C with 48 wt% P	Alloying	1 m NaClO_4_ in EC/DEC/FEC (1:1:0.2, v/v)	1250 at 1000 mA g^−1^ after 500 cycles	720.8 at 40 A g	[123]
36	RP@N‐doped carbon with 22.6 wt% P	Alloying	1 m NaClO_4_ in EC/DMC (1:1, v/v)	450 at 1 A g^−1^ after 1000 cycles	291 at 8 A g	[66]
37	RP@CNFs with 35 wt% P	Alloying	1 m NaClO_4_ in EC/DEC (1:1, v/v) with 10% FEC	≈1000 at 1 A g^−1^ after 5000 cycles (for P)	/	[17]
38	BP@C‐MWNT with 70 wt% P	Alloying	1 m NaPF_6_ in PC with 2% FEC	≈1700 at 1.3 A g^−1^ after 100 cycles (for P)	928 at 3 A g^−1^ (for P)	[124]
39	BP@RGO	Alloying	1 m NaClO_4_ in DMC with 10% FEC	1250 at 1 A g^−1^ after 500 cycles	720.8 at 40 A g	[69]
40	Few‐layer BP@PEDOT with 90.2 wt% P	Alloying	1 m NaClO_4_ in PC with 5% FEC	1078 at 0.1 A g^−1^ after 100 cycles	370 at 10 A g	[125]
41	Phosphorene	Alloying	1 m NaClO_4_ in PC with 5% FEC	≈1190 at 100 mA g^−1^ after 50 cycles	591 at 1.5 A g	[126]
42	Phosphorene@graphene with 48.3 wt% P	Alloying	1 m NaPF_6_ in EC/DEC (1:1, v/v) with 10%FEC	2080 at 50 mA g^−1^ after 100 cycles (for P)	645 at 26 A g^−1^ (for P)	[71]
43	Fe_3_O_4_@ graphene	Conversion	1 m NaClO_4_ in EC/DEC (1:1, v/v)	312 at 50 mA g^−1^ after 200 cycles	62 at 5 A g	[127]
44	Carbon confined Co_3_O_4_ nanoparticles	Conversion	1 m NaClO_4_ in PC/FEC (95:5, w/w)	409 at 0.5 A g^−1^ after 500 cycles	223 at 5 A g	[77]
45	SnO_2‐x_@CNFs	Conversion	1 m NaClO_4_ in EC/DEC (1:1, v/v)	565 at 1 A g^−1^ after 2000 cycles	340 at 5 A g	[128]
46	SnO_2_@ graphene	Conversion	1 m NaClO_4_ in EC/PC (1:1, v/v)	≈300 at 100 mA g^−1^ after 100 cycles	207 at 0.8 A g	[129]
47	Sb_2_O_3_	Conversion	1 m NaPF_6_ in EC/DEC/PC (4:4:2, v/v/v)	414 at 0.5 A g^−1^ after 200 cycles	265 at 5 A g	[78]
48	3D Ni‐supported Sb_2_O_3_	Conversion	1 m NaClO_4_ in PC with 5% FEC	397 at 0.2 A g^−1^ after 200 cycles	288 at 6.4 A g	[79]
49	Sn_4_P_3_@CNF	Conversion	1 m NaPF_6_ in EC/DMC (1:1, v/v) with 10% FEC	336 at 1 A g^−1^ after 500 cycles	321 at 5 A g	[82]
50	Sn_4_P_3_‐P@graphene	Conversion	1 m NaClO_4_ in EC/PC (1:1, v/v) with 10% FEC	≈550 at 1 A g^−1^ after 1000 cycles	315 at 10 A g	[130]
51	GeP_5_@C	Conversion	1 m NaClO_4_ in EC/DEC (1:1, v/v)	800 at 1000 mA g^−1^ after 110 cycles	380 at 5 A g	[131]
52	NiP_3_@CNT	Conversion	1 m NaClO_4_ in EC/DEC (1:1, v/v) with 5% FEC	≈682.4 at 200 mA g^−1^ after 120 cycles	363.8 at 1.6 mA g	[132]
53	FeP@carbon@ graphene	Conversion	1 m NaClO_4_ in EC/PC (1:1, v/v)	400 at 0.1 A g^−1^ after 250 cycles	237 at 1.6 A g	[133]
54	Ni_2_P immobilized on N, P‐co‐doped carbon nanosheets	Conversion	1 m NaClO_4_ in EC/DEC (1:1, v/v) with 5% FEC	181 at 500 mA g^−1^ after 1200 cycles	≈100 at 5 A g	[134]
55	Co_2_P@N‐doped carbon	Conversion	1 m NaClO_4_ in PC with 5% FEC	306 at 50 mA g^−1^ after 100 cycles	179 at 3 A g	[135]
56	MoS_2_@graphene	Conversion	1 m NaClO_4_ in EC/DMC (1:1, v/v)	421 at 300 mA g^−1^ after 250 cycles	201 at 50 A g	[83]
57	MoS_2_@carbon	Conversion	1 m NaClO_4_ in EC/PC (1:1, w/w) with 5% FEC	493.6 at 1 A g^−1^ after 500 cycles	401.3 at 10 A g	[136]
58	Few‐layer MoS_2_@carbon	Conversion	1 m NaPF_6_ in EC/DEC (1:1,v/v) with 5% FEC	443 at 1 A g^−1^ after 500 cycles	364 at 5 A g	[137]
59	Flower‐like MoS_2_@graphene	Conversion	1 m NaClO_4_ in EC/DEC (1:1, w/w) with 5% FEC	500 at 0.2 A g^−1^ after 100 cycles	345 at 1.6 A g	[86]

a) NaOTf: sodium triflate; DME: dimethoxyethane; EC: ethylene carbonate; DEC: diethyl carbonate; PC: propylene carbonate; FEC: fluoroethylene carbonate; DEGDME: diethylene glycol dimethyl ether; DMC: dimethyl carbonate; EMC: ethyl‐methyl carbonate; CNT: carbon nanotube; RGO: reduced graphene oxide; CNF: carbon nanofiber.

### 3.1 Insertion Anodes for SIBs

Due to the advantages of abundant reserves, low cost, and high mechanical strength, carbonaceous materials have been widely investigated as electrode materials for energy storage devices.^[^ ^14^, ^29^, ^30^ ^]^ Nowadays, graphite is the main anode materials for commercial LIBs, which have a mature commercial production line. However, it is disappointing that graphite is not suitable as anode materials for SIBs due to the weak interactions between Na^+^ and graphite.^[^ ^31^ ^]^ Stevens and Dahn demonstrated that most Na^+^ tended to deposit over the graphite surface rather than intercalating into the graphite layers during the sodiation process, thus leading to a negligible capacity.^[^ ^32^ ^]^ It was founded that only in ether‐based electrolytes, the solvated Na^+^ could be inserted into graphite layers, but the reversible capacity is only about 100 mAh g^−1^.^[^ ^33^, ^34^ ^]^

**Figure 3:**
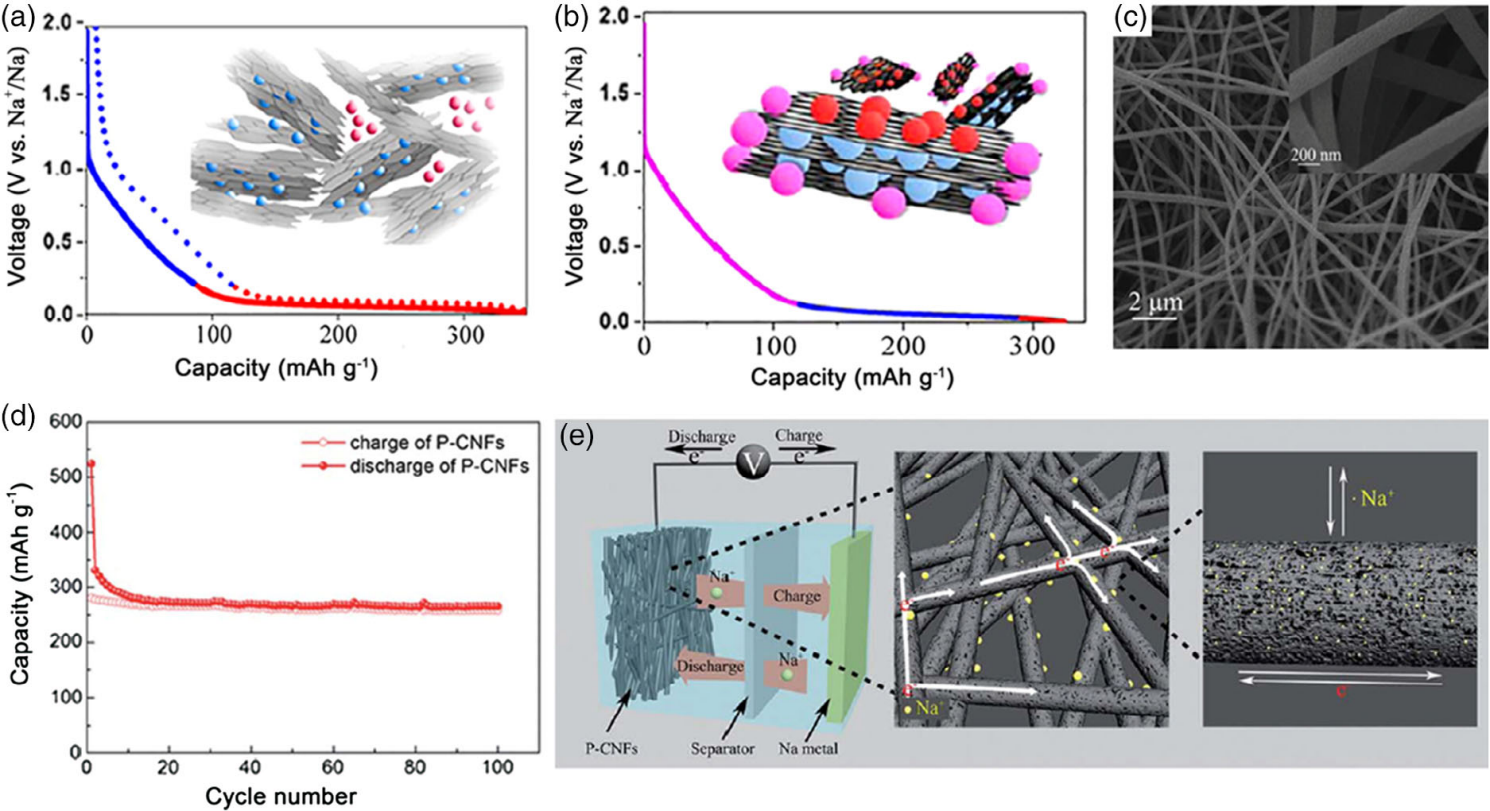
a) Potential diagram and schematic of the card‐house model on Na^+^ storage mechanism in hard carbon. The sloping region and plateau region represent the insertion of Na^+^ into defect assisted interlayer and micropore, respectively. b) Potential diagram and schematic of the three‐step process of Na^+^ storage mechanism in hard carbon. The sloping region, plateau region, and the end of plateau region represent the adsorption of Na^+^ at defective sites, insertion of Na^+^ into graphite layers, and adsorption of Na^+^ at the pore surface, respectively. a,b) Reproduced with permission.^[^ ^16^ ^]^ Copyright 2015, American Chemical Society. c) Scanning electron microscopy (SEM) image of P‐CNFs. d) Cycling performance of the P‐CNFs at 50 mA g^−1^. e) Schematic illustration of the Na^+^ storage mechanism of P‐CNFs. c–e) Reproduced with permission.^[^ ^42^ ^]^ Copyright 2014, Royal Society of Chemistry.

In the past decade, the research of carbonaceous materials as anode for SIBs has been mainly focused on hard carbon. In general, hard carbon principally consists of disordered structures, which cannot be graphitizable at high temperatures above 2500 °C.^[^ ^14^ ^]^ In 2000, Stevens and Dahn first reported the glucose‐derived hard carbon as anode for SIBs, which could deliver a reversible capacity of ≈300 mAh g^−1^.^[^ ^35^ ^]^ However, the low initial Coulombic efficiency (ICE) and poor rate capability dramatically hinder its further development. In 2011, Komaba et al. developed a hard carbon from the oxidized pitch, which showed good electrochemical performance as anode for SIBs.^[^ ^36^ ^]^ Since then, the research into hard carbon resumed, and a range of hard carbon materials have been widely investigated.^[^ ^37^, ^38^, ^39^, ^40^ ^]^ Among these studies, hard carbon shows the relatively high specific capacity ranging from 250 to 350 mAh g^−1^ as well as outstanding cycling stability, which made it an ideal first‐generation anode of choice for SIBs.^[^ ^1^, ^14^, ^41^ ^]^ As for the study on sodium storage mechanism of hard carbon materials, Stevens and Dahn first proposed a two‐step card‐house model, viz., defect‐assisted interlayer insertion of Na^+^ in the sloping region and intercalation into the micropore during the low voltage plateau, as shown in **Figure** **3**a.^[^ ^35^ ^]^ Later, Bommier et al. reported a totally different model, which can be summarized as a three‐step process: 1) the adsorption of Na^+^ at defective sites in the sloping region; 2) insertion of Na^+^ into graphite layers in the plateau region; and 3) adsorption of Na^+^ at the pore surface at the end of plateau region (Figure 3b).^[^ ^16^ ^]^ Up to now, the sodium storage mechanism of hard carbon is still debatable; thus, an in‐depth study should be conducted to further elucidate the process of inserting Na^+^ into hard carbon materials, which could be conducive to improve their sodium storage performance.

Recently, hard carbon materials with a nanostructure have provided new opportunities to elevate the electrochemical performance of SIBs due to the fairly good structural stability and connectivity for electrical conduction. For instance, through the electrospinning method and subsequent pyrolysis process, Yu and coworkers prepared the porous carbon nanofibers (P‐CNFs), which have an interconnected nanofiber structure with uniform diameter distribution (Figure 3c).^[^ ^42^ ^]^ As anode for SIBs, the P‐CNFs exhibited a reversible capacity of 331 mAh g^−1^ and retained 80.3% of the initial charge capacity after 100 cycles at 50 mA g^−1^ (Figure 3d). The good electrochemical performance can be attributed to the hierarchical porous channels, 3D interconnected structure, and good mechanical properties of the P‐CNFs, which could significantly shorten the transport distance for ions and electrons, reduce the contact resistances, and maintain excellent morphology stability, respectively (Figure 3e). Moreover, some studies have shown that the low capacity and poor ICE is significantly related to the large surface area of hard carbon materials, which could cause a more prominent formation of solid electrolyte interphase (SEI) layer during the initial discharge process and lead to a high first irreversible capacity. For example, Hu et al. prepared the wood cellulose fiber‐derived hard carbon and studied its electrochemical performance as anode for SIBs.^[^ ^43^ ^]^ They found that after pretreatment with 2,2,6,6‐tetramethylpiperidine‐1‐oxyl, the surface area of prepared hard carbon could effectively decrease from 586 to 126 m^2^ g^−1^. Due to the reduction of specific surface area, the ICE of the wood cellulose fibers derived hard carbon is significantly improved from 28% to 72%, as well as a stable cycling performance of 200 mAh g^−1^ at 100 mA g^−1^ after 200 cycles. In addition, to further improve the electrochemical performance of hard carbon materials, especially rate capability, the codoping with heteroatoms such as N, S, and P is an effective strategy.^[^ ^44^ ^]^ Recently, by carbonizing the citrate sodium and thiourea, Jiang and coworkers synthesized the hard carbon nanoparticles codoped with N, S elements (NSCs).^[^ ^45^ ^]^ The NSCs showed abundant defects and active covalent bonds, which could cause a pseudocapacitive behavior during the charge/discharge process, thus improving the electrochemical reaction kinetics. In addition, their study also indicated that the N, S codoping is more effective in increasing the specific capacity than single N atom doping due to the additional covalent S bonds and more defects. As a result, the optimized NSCs demonstrated outstanding rate capability, which exhibited a capacity of 280 mAh g^−1^ at 0.05 A g^−1^ and maintained 102 mAh g^−1^ at a high current density of 10 A g^−1^. Furthermore, this electrode also displayed stable cycling performance with a capacity of 223 mAh g^−1^ after 2000 cycles at 1 A g^−1^.

Compared with hard carbon, soft carbon has higher crystallinity and fewer defects, which can be graphitizable at high temperatures.^[^ ^14^ ^]^ It was demonstrated that soft carbon with enlarged interlayer distance could also be used as anode materials for SIBs.^[^ ^46^ ^]^ In general, soft carbon has a higher average oxidation voltage and lower capacity than hard carbon as anode for SIBs, which would lead to an inferior energy density of SIBs based on soft carbon anodes. However, due to the high conductivity caused by the high crystallinity carbons, soft carbon exhibits better rate capability than hard carbon, indicating potential applications in the field requiring high power density.^[^ ^47^ ^]^ Recently, Mai and coworkers developed microporous soft carbon nanosheets (SC‐NS) via a facile microwave‐assisted exfoliating process.^[^ ^29^ ^]^ Due to the porous structure and favorable defects on graphene layer edges, this SC‐NS electrode showed better electronic/ionic kinetics and extra storage sites than the conventional soft carbon microrod. The prepared SC‐NS displayed a reversible capacity of 232 mAh g^−1^ at 20 mA g^−1^ and 103.8 mAh g^−1^ at a high current of 1 A g^−1^ as anode for SIBs. Apart from the carbonaceous materials, some titanium‐based oxides also can store Na^+^ via the insertion reaction mechanisms.^[^ ^48^ ^]^ For example, TiO_2_ have been extensively studied as the insertion type anodes for SIBs due to the low cost and appropriate operating voltage. Recently, Zaccaria et al. reported the Mo‐doped anatase TiO_2_ coated by AlF_3_ and investigated its properties as anode for SIBs.^[^ ^49^ ^]^ Compared with the pristine TiO_2_, the Mo‐doped TiO_2_ shows better electronic/ionic conductivity and higher stability due to the Mo doping and AlF_3_ coating, which can exhibit a high capacity of 178.9 mA h g^−1^ at 0.1 C and outstanding rate capability up to 10 C.

Among the carbonaceous materials, hard carbon has been the most widely used anode for SIBs and shows good electrochemical performance; however, there are still some challenges in practical application. The storage mechanism of Na^+^ into hard carbon is still controversial; therefore, further research is needed to determine the sodium storage mechanism of hard carbon, which is helpful to theoretically guide the improvement of sodium storage performance of hard carbon. In addition, the inherent crystal structure of hard carbon leads to its poor rate capability as anode for SIBs. The strategies of heteroatom doping may solve this problems; thus, the effects of the type and content of doped elements on the electrochemical properties of hard carbon needs to be studied further. Furthermore, soft carbon shows better rate capability but lower capacity compared with hard carbon. It should be noted that the studies on soft carbon have been significantly less than that on hard carbon; therefore, it is expectable that better performance of soft carbon will be realized with continuing research.

### 3.2 Alloying Anodes for SIBs

The elements in groups 14 and 15 (including Sn, Sb, Bi, Ge, Pb, and P) can alloy with Na^+^ to form alloys and exhibit a theoretical capacity of 847, 660, 385, 369, 485, and 2596 mAh g^−1^, respectively, indicating high potential applications as anode materials for SIBs.^[^ ^25^, ^50^ ^]^ In 1987, Vernick and coworkers first reported the Na cell based on the alloying reaction mechanism using a Pb composite electrode.^[^ ^51^ ^]^ In the following few decades, numerous metal anode materials for SIBs have been studied. Compared with the materials based on the insertion reaction mechanism, the alloying‐type materials can interact with more Na^+^, thus generating a higher capacity. However, a huge volumetric change would be involved during the alloying process of these materials, which would dramatically reduce the lifespan of batteries.^[^ ^52^ ^]^ For example, the volumetric change of Sn can be as high as 420% by alloying with Na^+^ to form a Na_15_Sn_4_ binary compound. Such high volumetric change would make the electrode materials suffer from high mechanical stress, which would cause the pulverization of electrodes, leading to a rapid capacity fading.^[^ ^15^, ^53^ ^]^ To solve this problem, lots of effective strategies have been used on modifying the alloying anodes, and some of the recent progress are discussed in the following text.

#### 3.2.1 Sn‐Based Anodes for SIBs

**Figure 4:**
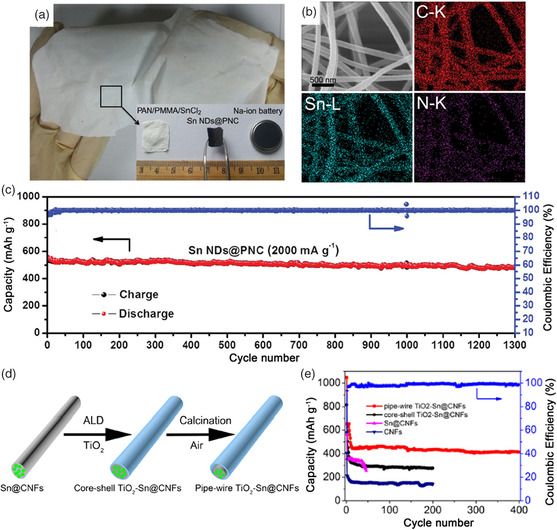
a) Digital photos of the as‐spun polyacrylonitrile/polymethyl methacrylate/SnCl_2_ (PAN/PMMA/SnCl_2_) film and the free‐standing Sn NDs@PNC electrode for SIBs. b) EDS mapping of Sn NDs@PNC nanofibers. c) Cycling performance of Sn NDs@PNC at 2 A g^−1^ for 1300 cycles. a–c) Reproduced with permission.^[^ ^56^ ^]^ Copyright 2015, Wiley-VCH. d) Schematic illustration of the preparation procedure of pipe‐wire TiO_2_‐Sn@CNFs. e) Cycling performance of pipe‐wire TiO_2_‐Sn@CNFs, core–shell TiO_2_‐Sn@CNFs, Sn@CNFs, and CNFs at 100 mA g^−1^. d,e) Reproduced with permission.^[^ ^57^ ^]^ Copyright 2017, American Chemical Society.

In 2012, Komaba et al. pioneered the work of examining the electrochemical performance of Sn as anode for SIBs, which delivered a high capacity of 500 mAh g^−1^.^[^ ^54^ ^]^ However, due to the severe volumetric change caused by the repeating transformation between Sn and Na–Sn alloy phases, this SIB showed a poor cycling ability for only 20 cycles. The electrochemical alloying process of Sn at room temperature mainly occurs in a two‐step reaction, viz., amorphous Na_*x*_Sn (*x* ≈ 0.5), and crystalline Na_15_Sn_4_, as reported by Huang and coworkers^[^ ^53^ ^]^ During the first step, by alloying with Na^+^ to form Na‐poor Na_*x*_Sn, the max volumetric change of Sn is only about 60%. When the amorphous Na_*x*_Sn converts into crystalline Na_15_Sn_4_ after full sodiation in the second step, the Sn anode can offer a high theoretical capacity of 847 mAh g^−1^; however, the volumetric expansion could also reach as high as 420%. The huge volumetric changes would crack the electrodes, which would cause the active material to lose electrochemical contact with the current collector, thus leading to rapid capacity decay. To deal with this, the uniform distribution of Sn particles into a conductive carbon matrix is an effective method. By adopting this strategy, the volumetric expansion of Sn during the sodiation process can be inhibited to some extent; however, the agglomeration of Sn particles becomes another limitation of the cycling performance.^[^ ^55^ ^]^ Therefore, it is important to design and optimize the structure of Sn–carbon composite. For instance, Chen and coworkers designed porous N‐doped carbon nanofibers (denoted as Sn NDs@PNC) by a simple electrospinning technique and subsequent pyrolysis.^[^ ^56^ ^]^ The NDs@PNC displayed excellent flexibility and could be directly used as an anode without an extra binder and current collector (**Figure** **4**a). In addition, the energy dispersive spectrometer (EDS) mapping (Figure 4b) reveals that the Sn and N are uniformly distributed along with carbon matrix. As anode for SIBs, the Sn NDs@PNC delivered a high reversible capacity of 633 mAh g^−1^ at 200 mA g^−1^ and 450 mAh g^−1^ at 10 A g^−1^, indicating superior rate capability. It also exhibited excellent cycling stability, with a capacity of 484 mAh g^−1^ after 1300 cycles at 2 A g^−1^ (Figure 4c). The excellent electrochemical performance can be attributed to the ultrasmall Sn nanodots and 3D conductive network formed by the nitrogen‐doped carbon nanofibers, which not only contributes to the diffusion kinetics of electrons and Na^+^ but also buffers the large volume fluctuations of Sn nanodots, as well as preventing its aggregation during the cycling process. Wang and coworkers synthesized a TiO_2_–Sn@carbon nanofibers composite (TiO_2_–Sn@CNFs) with a pipe‐wire structure by combining electrospinning with the atomic layer deposition method (Figure 4d).^[^ ^57^ ^]^ The TiO_2_ shell distributed along the Sn@CNFs axis could further restrain the volume variation of Sn nanoparticles during the cycling process. As a result, this pipe‐wire TiO_2_‐Sn@CNFs delivered an enhanced electrochemical performance, with a high reversible capacity of 413 mAh g^−1^ at 100 mA g^−1^ after 400 cycles (Figure 4e). Apart from the 2D Sn–carbon composite, 3D Sn–carbon composite also has a good effect in relieving the volumetric expansion and creating extra electronic transmission channels. For example, Wang and coworkers fabricated a 3D hierarchical architecture electrode with Sn@carbon nanotube (CNT) nanopillars grown vertically on carbon paper (Sn@CNT‐CP).^[^ ^58^ ^]^ The unique nanoforest architecture of the Sn@CNT could effectively alleviate the volumetric expansion effect in the alloying process and facilitate the transmission of both electrons and Na^+^. As a result, this Sn@CNT‐CP anode delivered high reversible capacity, good rate capability, as well as an extended cycle life to 100 cycles.

#### 3.2.2 Sb‐Based Anodes for SIBs

**Figure 5:**
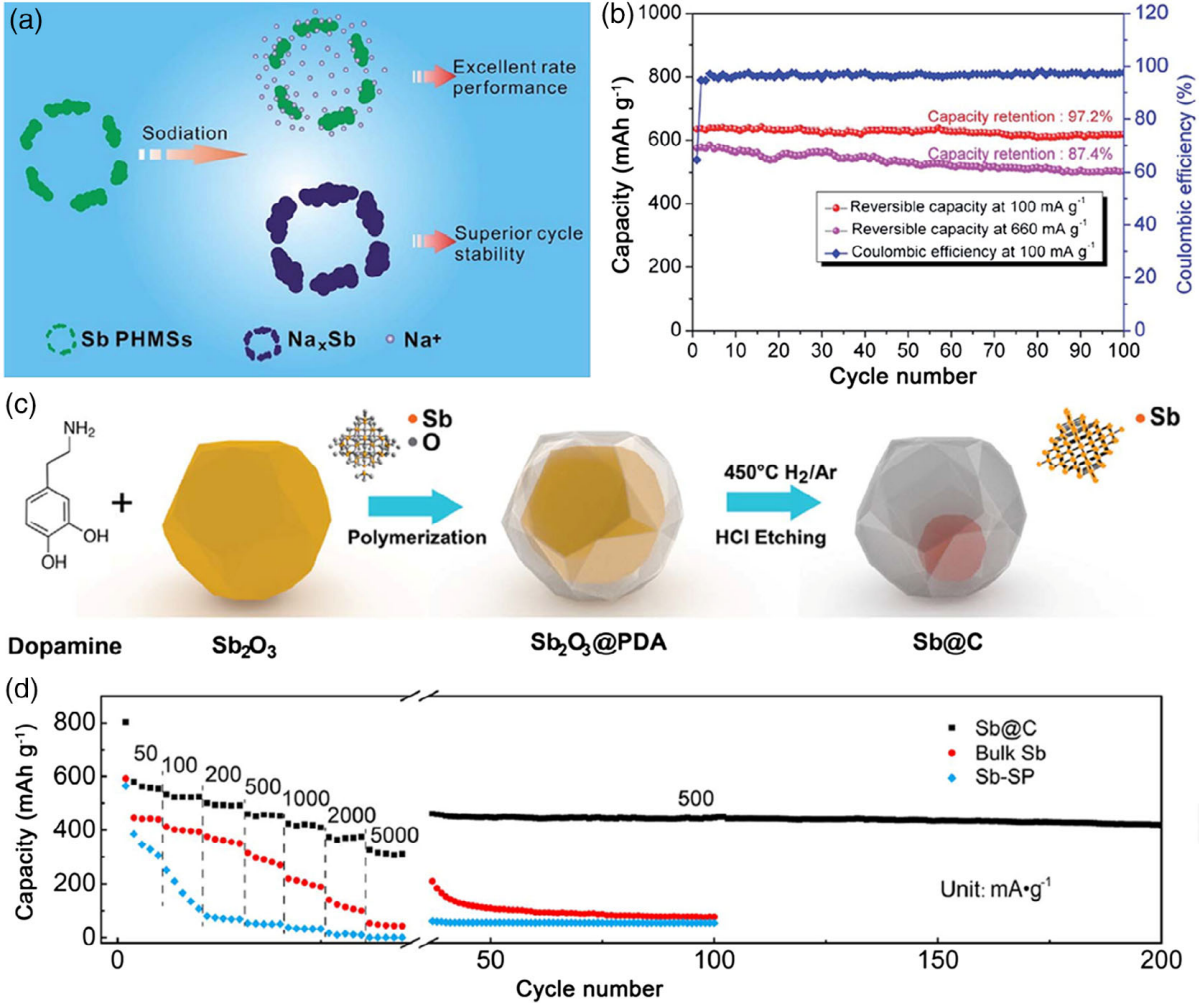
a) Schematic illustration of the sodiation process of Sb PHMS, indicating excellent rate performance and superior stability. b) Cycling performance of Sb PHMS at current densities of 100 and 660 mA g^−1^, respectively. a,b) Reproduced with permission.^[^ ^63^ ^]^ Copyright 2015, Royal Society of Chemistry. c) Schematic illustration of the preparation procedure of Sb@C composite. d) Comparison of rate and cycling performance of Sb@C, bulk Sb, and Sb‐super P (Sb-SP) as anode for SIBs. c,d) Reproduced with permission.^[^ ^62^ ^]^ Copyright 2017, Elsevier.

Sb is also a promising anode material for SIBs, which can alloy with Na^+^ to form Na_3_Sb and release a high theoretical capacity of 660 mAh g^−1^.^[^ ^59^, ^60^ ^]^ Using the in situ X‐ray powder diffraction (XRD) characterization method, Monconduit and coworkers investigated the electrochemical alloying mechanism between Sb and Na^+^.^[^ ^61^ ^]^ They found that the crystalline Sb would alloy with Na^+^ to form amorphous Na_*x*_Sb (*x* ≈ 1.5) at first; Then, with the continuous insertion of Na^+^, amorphous Na_*x*_Sb would ultimately transform to hexagonal Na_3_Sb. During the sodiation process, Sb would also suffer from a huge volumetric change (390% for Na_3_Sb), thus resulting in electrode pulverization and rapid capacity fading. It has been proved that the construction of Sb–carbon composite and tuning morphology at the nanoscale, such as porous nanospheres, nanofibers, nanotubes, nanorods, and yolk–shell structures, can effectively mitigate the volumetric change of Sb anode.^[^ ^59^, ^62^, ^63^, ^64^ ^]^ Ji and coworkers prepared the Sb porous hollow microspheres (PHMSs) through a template sacrifice method using Zn microspheres as a template.^[^ ^63^ ^]^ The hollow internal structure of Sb PHMS can provide extra space to accommodate the volumetric expansion, thus leading to superior cycle stability. Meanwhile, the rough porous shell can offer numerous channels for the diffusion of Na^+^, which could dramatically facilitate the reaction kinetics, leading to excellent rate performance (**Figure** **5**a). As a result, the Sb PHMS anode delivered a high reversible capacity of 617 mAh g^−1^ at 100 mA g^−1^, outstanding rate capability of 312.9 mAh g^−1^ at 3.2 A g^−1^ and remained a capacity of 97.2% at 100 mA g^−1^ after 100 cycles (Figure 5b). To elevate the conductivity and accommodate the volume expansion of electrodes, Paik and coworkers synthesized the Sb@C coaxial nanotubes through a simple carbon‐coating and thermal‐reduction method.^[^ ^59^ ^]^ The prepared Sb@C displayed excellent rate capability of 310 mAh g^−1^ at 20 A g^−1^ and impressively ultralong cycle life for 2000 cycles. Li et al. designed the yolk–shell‐structured Sb@C composite by a selective reduction and etching method (Figure 5c).^[^ ^62^ ^]^ The unique yolk–shell structure can accommodate the volumetric expansion of Sb during the sodiation process. Even if Sb breaks during the cyclic process, the debris will remain in the carbon shell without contact with the electrolyte, which could avoid the infaust side reactions. As a result, the Sb@C anode delivered a high reversible capacity of 554 mAh g^−1^ at 50 mA g^−1^ and stable cycling performance for over 200 cycles (Figure 5d).

#### 3.2.3 P‐Based Anodes for SIBs

**Figure 6:**
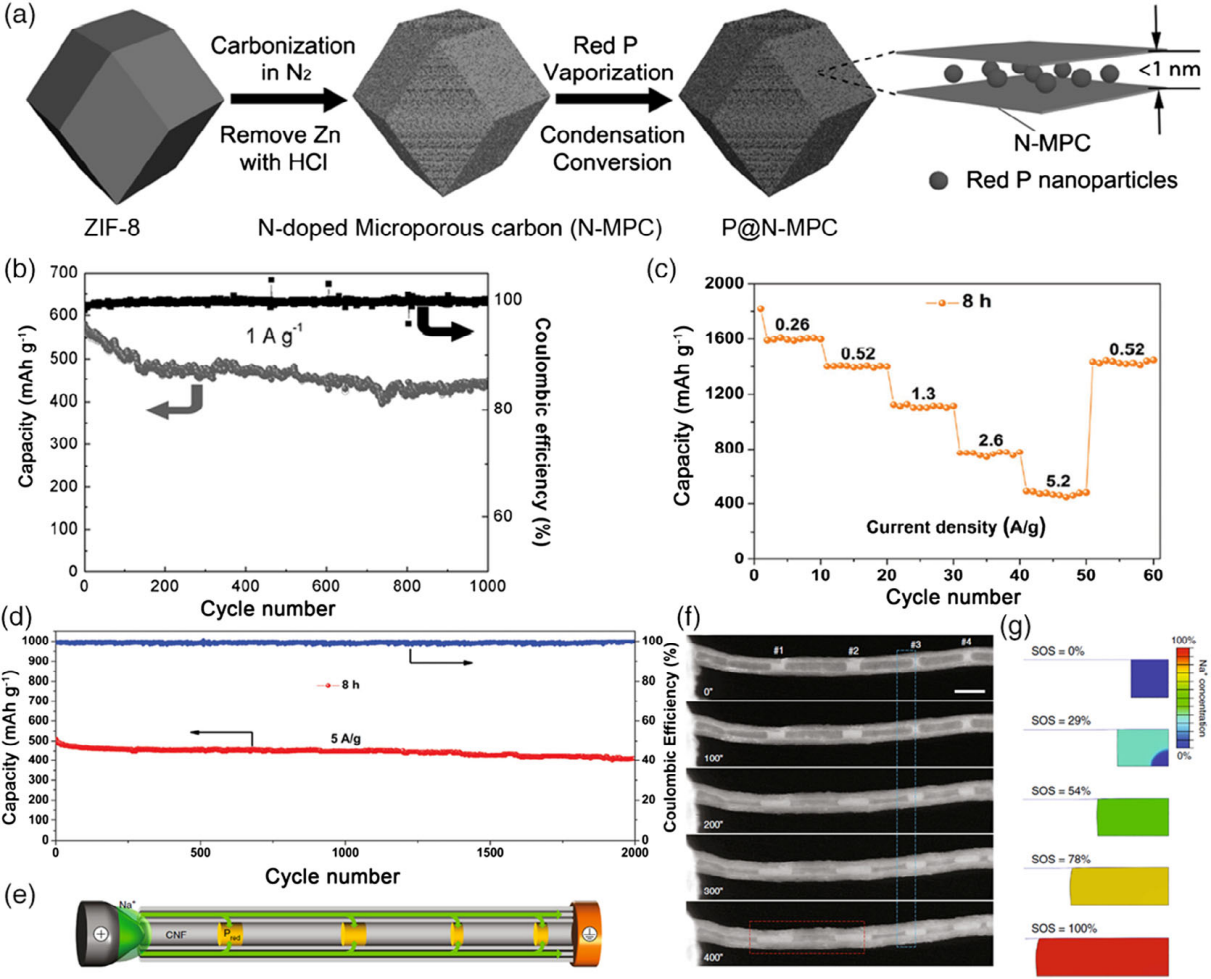
a) Schematic illustration of the preparation process of P@N‐MPC. b) Cycling performance of P@N‐MPC at 1 A g^−1^. Reproduced with permission.^[^ ^66^ ^]^ Copyright 2017, Wiley. c) Rate capability of RP@Ni‐P at different current densities ranging from 0.26 to 5.2 A g^−1^. d) Cycling performance of RP@Ni‐P at 5 A g^−1^. Reproduced with permission.^[^ ^67^ ^]^ Copyright 2017, Royal Society of Chemistry. e) Schematic illustration of the in situ TEM setup of a dry electrochemical cell. f) STEM images captured from the in situ TEM measurement in real‐time revealing the volumetric change of RP (scale bar corresponds to 200 nm). g) Simulated state of sodiation (SOS) on the basis of the RP morphological evolution from the blue rectangle in (f). Reproduced with permission.^[^ ^17^ ^]^ Copyright 2020, Springer Nature.

There are mainly three allotropes of phosphorus, i.e., white phosphorus (WP), red phosphorus (RP), and black phosphorus (BP). Due to its highly volatile, active, and toxic physicochemical properties, WP is not suitable as an electrode material for batteries. RP is naturally abundant, environmentally friendly, and has a super high theoretical capacity (2596 mAh g^−1^), which is regarded as one of the most promising anode candidates for SIBs. However, the low electronic conductivity (≈10^−14^ S cm^−1^) and huge volumetric change (440%) dramatically hinder its electrochemical performance.^[^ ^65^ ^]^ Combining active materials with carbon is a traditional but still effective technique to enhance electrical conductivity, facilitate Na^+^ diffusion, and maintain structural integrity. Yu et al. prepared the RP@carbon composite by confining nanosize amorphous RP into zeolitic imidazolate framework‐8‐derived nitrogen‐doped microporous carbon matrix (P@N‐MPC), as shown in **Figure** **6**a.^[^ ^66^ ^]^ As anode for SIBs, this P@N‐MPC exhibited a high reversible capacity of 600 mAh g^−1^ at 0.15 A g^−1^, and a stable capacity of 450 mAh g^−1^ at 1 A g^−1^ after 1000 cycles with a capacity retention of 80% (Figure 6b). The good performance can be attributed to the highly nitrogen‐doped microporous carbon, which could simultaneously build up a highly conductive pathway for the transmission of electrons and stabilize RP during the sodiation process. Moreover, the uniform micropores can confine RP to nanosize, which could relieve the strain from volumetric change. In addition to bonding with conductive carbon, combining phosphorus with metals also works well. For instance, An et al. synthesized the RP@Ni‐P core@shell nanostructures by combining electroless deposition with a chemical dealloying technique.^[^ ^67^ ^]^ The in situ generated Ni_2_P on the RP particle surface can promote intimate contact between RP and the Ni‐P shell, leading to a strong electrode structural integrity. In addition, the amorphous Ni–P outer shell can enhance the charge transfer, ensuring ultra‐fast electron transport of the composite. As a result, the RP@Ni‐P anode exhibited good rate capability of 491 mAh g^−1^ at 5.2 A g^−1^ and an ultralong cycling performance over 2000 cycles (Figure 6c,d). So far, the sodium storage performance of RP has been greatly enhanced; however, the evidence uncovering the mechanism of its capacity fading is currently indistinct. Recently, Zhou et al. prepared a RP‐impregnated carbon nanofiber composite (P@CNF) and investigated its sodiation process and capacity fading mechanism through an in situ TEM technique and chemo‐mechanical simulation (Figure 6e–g).^[^ ^17^ ^]^ They found that RP particles would be softened in the alloying process, which showed excellent malleability. Furthermore, the main reason for the capacity decay of RP could be attributed to the side reactions that occurred during the sodiation process in which the extremely reactive sodiated phosphorus compounds would form. The prepared P@CNF exhibited a high reversible capacity of ≈1850 mAh g^−1^ at 0.1 A g^−1^ and ultralong cycling life over 5000 cycles at 1 A g^−1^.

BP is the most thermodynamically stable allotrope of phosphorus, which has one amorphous form and three crystalline modifications (orthorhombic, rhombohedral, and cubic) caused by different temperatures and pressures.^[^ ^68^ ^]^ Due to the superior electrical conductivity (10^2^ S cm^−1^), BP normally shows better sodium storage performance than RP.^[65a]^ However, the lack of effective and low‐cost synthesis methods to produce BP on a large scale has hindered its development as anodes for high‐performance SIBs. With respect to this, a facile and scalable method by application of pressure (8 GP) at room temperature to prepare black phosphorus/reduced graphene oxide composite (BP/RGO) was developed by Zhou et al.^[^ ^69^ ^]^ The highly conductive BP and graphene can simultaneously facilitate Na^+^ and electron transfer kinetics. Moreover, the graphene network can provide robust mechanical support, which could buffer the huge volumetric change of BP. As a result, the BP/RGO displayed a superior rate capability of 720.8 mAh g^−1^ at 40 A g^−1^ and a long cycling lifespan over 500 cycles. In addition, By stripping the bulk BP into one or few layers through physical or chemical methods, the so‐called phosphorene with a 2D structure can be obtained. Compared with BP, the interlayer spacing, specific surface area, electronic properties, and Na^+^ diffusivity of phosphorene are dramatically enhanced, leading to outstanding electrochemical performance.^[^ ^70^ ^]^ For instance, Cui and coworkers synthesized a phosphorene–graphene composite with a sandwich‐like structure by the liquid‐phase exfoliation method.^[^ ^71^ ^]^ In this composite, the graphene layers can provide an elastic buffer to alleviate the effect of volumetric expansion, and the phosphorene layers can short the diffusion length for Na^+^. The prepared phosphorene–graphene composite delivered a high specific capacity of 2440 mAh g^−1^ at 50 mA g^−1^ and maintained 83% of the initial capacity after 100 cycles.

In summary, the huge volumetric expansion of the electrode during the sodiation process is the main challenge for the anodes based on alloying reaction mechanisms. The current researches on these alloying‐type materials mainly focus on the construction of nanostructured composites to accommodate the volumetric expansion, which can significantly improve the electrochemical performance of electrode materials, such as cycling performance and rate capability. However, it should be noted that the construction of nanostructured composites will reduce the load of active material on the current collector, as the compaction density of nanomaterials is low, which is not conducive to the practical large‐scale applications.

### 3.3 Conversion Anodes for SIBs

Due to the high capacity and relatively long cycling life, metal oxides/phosphides/sulfides have attracted more and more attention as anode for SIBs. During the sodiation process, these materials will undergo a conversion reaction to form a new compound by chemical transformation, which can be categorized into conversion‐type materials. In addition, depending on the type of metallic elements, some of these materials would undergo an insertion or alloying process along with the conversion process. The conversion‐type anodes show higher capacity than the insertion type anodes and less volumetric change than the alloying‐type anodes, indicating good application prospect.^[^ ^72^ ^]^ Here, we concentrated mainly on several typical materials reported in recent years.

#### 3.3.1 Metal Oxides Anodes for SIBs

**Figure 7:**
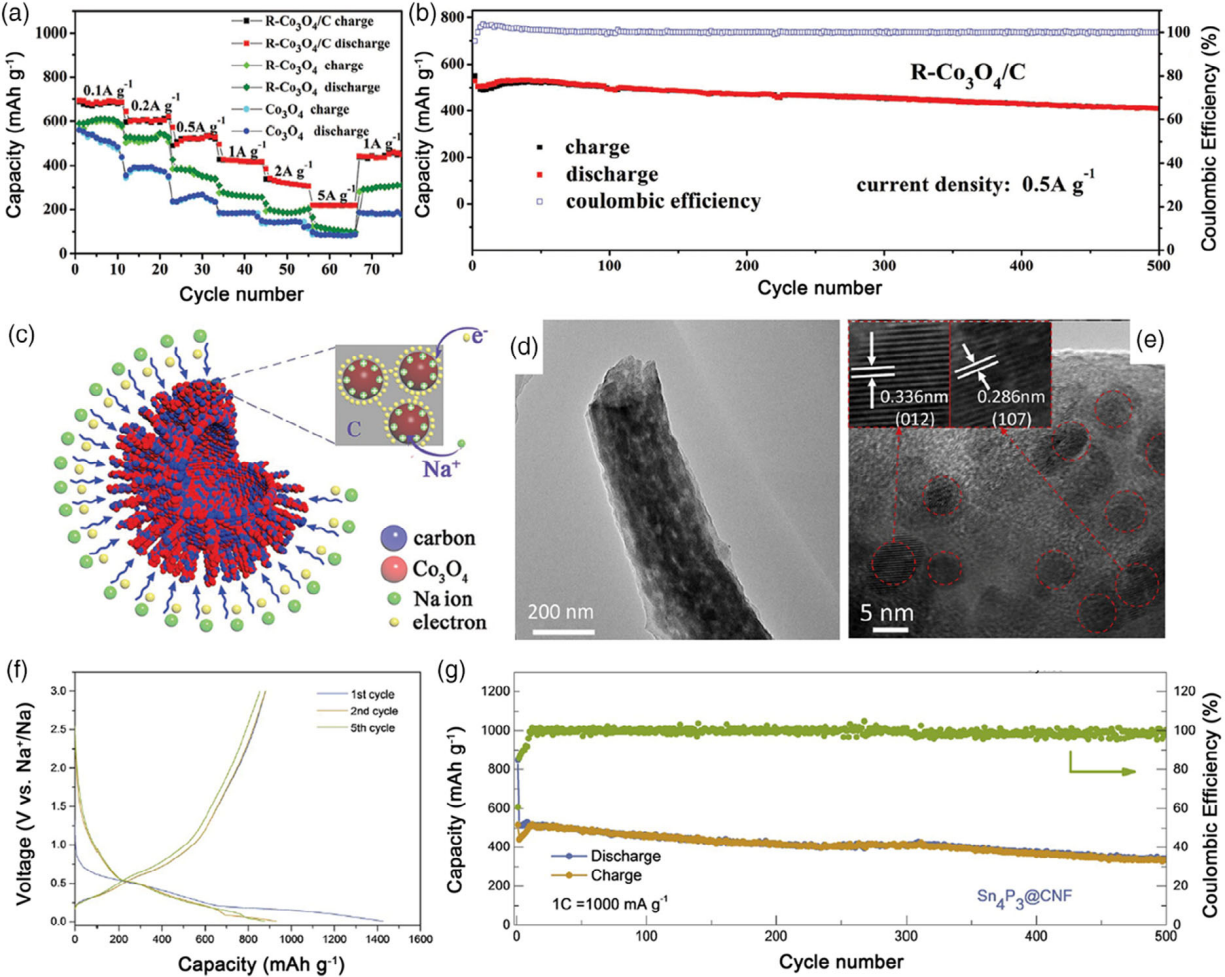
a) Rate capability of Co_3_O_4_, R‐Co_3_O_4_, and R‐Co_3_O_4_/C at different current densities ranging from 0.1 to 5 A g^−1^. b) Cycling performance of R‐Co_3_O_4_/C at 0.5 A g^−1^. c) Schematic illustration of the advantages of R‐Co_3_O_4_/C during the sodiation process. a–c) Reproduced with permission.^[^ ^77^ ^]^ Copyright 2018, Wiley-VCH. d) TEM, e) High-resolution TEM (HRTEM) images of Sn_4_P_3_@CNF. f) Galvanostatic discharge/charge profiles of Sn_4_P_3_@CNF at 100 mA g^−1^. g) Cycling performance of Sn_4_P_3_@CNF at 1 A g^−1^. d–g) Reproduced with permission.^[^ ^82^ ^]^ Copyright 2020, Elsevier.

According to the different electrochemical reaction mechanisms, metal oxides can be divided into transition metal oxides (TMOs) and alloy metal oxides. For TMOs, the transition metallic element, such as Co, Fe, Cu, Ni, and Zn, is electrochemically inactive in the oxides, leading to a one‐step conversion reaction between the TMOs and Na^+^.^[^ ^73^ ^]^ Tirado and coworkers first reported the TMOs of NiCo_2_O_4_ as anode for SIBs, which delivered a reversible capacity of 200 mAh g^−1^.^[^ ^74^ ^]^ After that, many other TMOs, such as Co_3_O_4_, Fe_2_O_3_, Fe_3_O_4_, MnO, and CuO, have been intensively studied.^[^ ^75^ ^]^ Due to the high theoretical capacity (890 mAh g^−1^), Co_3_O_4_ has been widely studied as anode materials for SIBs; however, the poor electrical conductivity and sluggish kinetics during the sodiation process significantly hinder its practical application.^[^ ^76^ ^]^ Recently, Xia and coworkers prepared the rambutan‐like carbon confined Co_3_O_4_ nanoparticles hybrid hollow spheres (R‐Co_3_O_4_/C) via a one‐pot hydrothermal treatment method.^[^ ^77^ ^]^ Compared with pure Co_3_O_4_, the R‐Co_3_O_4_/C electrode displayed a high reversible capacity of 712 mAh g^−1^ at 0.1 A g^−1^ and sustained 223 mAh g^−1^ at 5 A g^−1^, as well as stable cycling performance for over 500 cycles, as shown in **Figure** **7**a,b. The improved electrochemical performance could be attributed to the 3D hollow carbon ball structure, which not only buffered the volumetric change but also enhanced the transfer of both electrons and Na^+^ (Figure 7c).

In addition to TMOs, the alloy metal oxides, such as SnO_2_ and Sb_2_O_3_, would react with Na^+^ through a conversion reaction at the first step. Because the metallic element is electrochemically active in the alloy metal oxides, a subsequent alloying reaction would occur after the conversion reaction. Due to the relatively stable cycling performance, Sb_2_O_3_ has been widely investigated as anode materials for SIBs. Yan and coworkers first demonstrated that Sb_2_O_3_ inserted/extracted Na^+^ via a conversion–alloying mechanism.^[^ ^78^ ^]^ Their research indicated that the crystal Sb_2_O_3_ could initially store Na^+^ to form Na_*x*_Sb_2_O_3_ in the voltage range of 0.6–1.0 V. Then, the formed Na_*x*_Sb_2_O_3_ would keep reacting with Na^+^ to form Sb and Na_2_O via a conversion mechanism. This conversion reaction in the subsequent cycles would be conducted with amorphous Sb_2_O_3_ due to the breakdown of the cubic structure of Sb_2_O_3_ in the first cycle. With the continuation of the discharge process, Sb would react with Na^+^ to form the NaSb phase via an alloying mechanism. The reaction mechanism of Sb_2_O_3_ with Na^+^ could be summarized as follows

(1) $${\text{Sb}}_{2}O_{3}+x{\text{Na}}^{+}+xe^{-1}\to {\text{Na}}_{x}{\text{Sb}}_{2}O_{3}\;(1\text{st discharge})$$

(2) $${\text{Na}}_{x}{\text{Sb}}_{2}O_{3}+(6-x){\text{Na}}^{+}+(6-x)e^{-1}\to 2\text{Sb}+3{\text{Na}}_{2}O\;(1\text{st discharge})$$

(3) $${\text{Sb}}_{2}O_{3\text{(amorphous)}}+6{\text{Na}}^{+}+6e^{-}↔2\text{Sb}+3{\text{Na}}_{2}O\;(\text{after}1\text{st cycle})$$

(4) $$\text{Sb}+{\text{Na}}^{+}+e^{-}↔\text{NaSb}$$

The in situ formed Na_2_O during the conversion reaction process can act as a buffer matrix to alleviate the volumetric change and improve the cycling stability. As a result, the Sb_2_O_3_ anode exhibited stable cycling performance for over 200 cycles. Although conversion–alloying‐type materials exhibit relatively good cycling performance than pure alloying materials, they still suffer from the volumetric change mainly caused by the subsequent alloying process. Fabrication of nanoarchitecture materials or combining with 2D or 3D matrix may be an effective way for further improving the electrochemical performance. Recently, Braun and coworkers synthesized a 3D Ni‐supported NiSb/Sb_2_O_3_ electrode (Ni@NiSb/Sb_2_O_3_) through a pulsed electrodeposition method and subsequent heat treatment.^[^ ^79^ ^]^ The 3D Ni scaffold could effectively accommodate the volumetric change caused by Sb_2_O_3_ during the alloying process, thus leading to better cycling performance. Moreover, during the heat treatment process, a NiSb alloy formed between the 3D Ni scaffold and Sb_2_O_3_, which could enhance the adhesion of Sb_2_O_3_ to the 3D Ni scaffold. As a result, this 3D Ni@NiSb/Sb_2_O_3_ achieved a high capacity of 445 mAh g^−1^ at 200 g^−1^ and retained a capacity retention of 89% after 200 cycles.

#### 3.3.2 Metal Phosphides Anodes for SIBs

Compared with phosphorus, metal phosphides display higher electrical conductivities due to the presence of metal atoms.^[^ ^80^ ^]^ For example, Sn_4_P_3_ can exhibit a high electronic conductivity of 30.7 S cm^−1^ (≈10^−14^ S cm^−1^ for RP) and release a high theoretical volumetric specific capacity of 6650 mAh cm^−3^ (5710 mAh cm^−3^ for RP), which have attracted considerable attention as promising anode for SIBs. In 2014, Lee and coworkers pioneered the work of studying the sodium storage capability of Sn_4_P_3_.^[^ ^81^ ^]^ During the discharge process, Sn_4_P_3_ would react with Na^+^ via a conversion–alloying reaction to form Na_3_P and Na_15_Sn_4_, which could exhibit a reversible capacity of 718 mAh g^−1^ and stable cycling performance for over 100 cycles. In addition, Sn_4_P_3_ displayed a more appropriate low redox potential than RP (0.3 V vs Na/Na^+^ for Sn_4_P_3_ and 0.5 V vs Na/Na^+^ for RP), indicating good application prospect in the full cell. Recently, Ran et al. prepared a Sn_4_P_3_@CNF composite by electrospinning method.^[^ ^82^ ^]^ In this composite, ultrasmall Sn_4_P_3_ particles (≈8 nm) were encapsulated into porous carbon nanofibers (Figure 7d,e), which could allow the electrolytes to easily penetrate into the nanofibers and effectively accommodate the volumetric change caused by Sn_4_P_3_ during the sodiation process, leading to more electrochemical reaction sites and long cycling life. As a result, the Sn_4_P_3_@CNF displayed a high reversible capacity of 930 mAh g^−1^ at 100 mA g^−1^ and stable cycling performance for over 500 cycles (Figure 7f,g).

#### 3.3.3 Metal Sulfides Anodes for SIBs

Among the metal sulfides, MoS_2_ has drawn considerable attention as an anode for LIBs due to its high reversible capacity. In MoS_2_ materials, Mo and S atoms are covalently bonded to form MoS_2_ layer, and the adjacent layers are stacked through van der Waals interactions, showing an interlayer spacing of about 6.2 Å, which is also favorable for the intercalation of larger Na^+^ in addition to Li^+^.^[^ ^83^, ^84^ ^]^ Chen and coworkers founded that during the sodiation process, Na^+^ will first insert into the layered structure of MoS_2_ above 0.4 V, and then the conversion reaction would occur below 0.4 V as follows^[^ ^85^ ^]^

(5) $$\text{Intercalation reaction}:\;{\text{MoS}}_{2}+x\text{Na}={\text{Na}}_{x}{\text{MoS}}_{2}\;(\text{above}0.4\;\text{V vs Na}/{\text{Na}}^{+})$$

(6) $$\text{Conversion reaction}:\;{\text{Na}}_{x}{\text{MoS}}_{2}+(4-x)\text{Na}=\text{Mo}+2{\text{Na}}_{2}S\;(\text{below}\;0.4\;\text{V vs Na}/{\text{Na}}^{+})$$

The conversion reaction process below 0.4 V would cause volumetric change, leading to poor cycling stability. Adjusting the voltage window above 0.4 V can avoid the conversion reaction process, thus improving the cycling performance; however, the capacity would also dramatically be reduced. In addition, the low electronic conductivity between the adjacent S–Mo–S sheets also compromises the electrochemical performance of MoS_2_. Thus, it is important to make innovations in materials architecture design. Due to the high electrical conductivity and excellent mechanical properties, graphene is often used to combine with MoS_2_ for better electrochemical performance.^[^ ^83^, ^84^, ^86^, ^87^ ^]^ For example, using bulky MoS_2_ and graphite, Wang and coworkers synthesized MoS_2_/graphene nanosheets.^[^ ^83^ ^]^ As anode for SIBs, the MoS_2_/graphene showed remarkably high rate capability of 284 mAh g^−1^ at 20 A g^−1^ and stable cycling performance, with 95% capacity retention at 0.3 A g^−1^ after 250 cycles. Inspired by natural marigold flowers, Anwer et al. designed a 3D ultrathin flower‐like microstructure composed of MoS_2_ nanoflowers and layered graphene (MoS_2_‐G) via a controlled hydrothermal method.^[^ ^86^ ^]^ The ultrathin nature of this composite can deliver enhanced electrical conductive channels. In addition, the flower‐like structure and layered graphene wrapping can accommodate the volumetric variation of MoS_2_ during the sodiation process. As a result, the MoS_2_‐G displayed a high reversible capacity of 606 mAh g^−1^ at 200 mA g^−1^ and remarkable rate capability of 345 mAh g^−1^ at 1600 mA g^−1^.

In general, combining active materials based on alloying and conversion reaction with carbonaceous materials is an effective technique to promote the Na^+^ diffusion, create the electric conducting pathways, and buffer the volumetric strain. However, despite the enormous advantages, there are still some issues with using such approaches in practical applications. For example, compositing with carbonaceous materials can lead to a low ICE of the batteries due to the more SEI formation on the carbon surface, which would cause a high irreversible capacity loss during the initial cycles. Apart from carbon only offering a small amount of capacity compared with the active materials, the high carbon content will dramatically lower the energy density of the batteries. Therefore, to get better electrochemical performance, we should balance the carbon amount with active materials and optimize the electrolytes, as well as the structural design of electrodes.

## 4 Anodes for SDIBs

As a new type of energy storage device, SDIBs have come to the attention of researchers in recent years.^[^ ^18^, ^88^ ^]^ However, due to lack of applicable anode materials for Na^+^ insertion, the development of high‐performance SDIBs still remains a great challenge. Up to now, only a few kinds of anode materials have been investigated in SDIBs system. In principle, the materials that could store Na^+^ in SIBs can also be used as anodes for SDIBs. However, different from the reaction mechanism of SIBs, Na^+^ and anions react simultaneously with anode and cathode, respectively, in SDIBs, so the kinetic matching problem of anode and cathode needs to be considered. Therefore, the exploration of suitable anode materials plays an important role in the construction of SDIBs. In this section, the typical recently reported anodes for SDIBs, including insertion‐type materials, alloying‐type materials, and conversion‐type materials, are discussed. The detailed electrochemical performance of various reported SDIBs in recent years is shown in **Table** **2**.

**Table 2:** Summary of electrochemical performances of recent progress on SDIBs

No	Anode||cathode materialsa)	Reaction mechanism of anodes	Electrolytes	Cycling performance [mAh g^−1^]	Rate capability [mAh g^−1^]	References
1	Hard carbon||KS_6_ graphite	Insertion	1 m NaPF_6_ in EC/EMC (1:2, v/v)	127 at 500 mA g^−1^ after 1000 cycles	98 at 1000 mA g	[89]
2	Hard carbon||graphite	Insertion	0.8 m NaPF_6_ in PC	≈53 at 186 mA g^−1^ after 200 cycles (cathode)	46 at 558 mA g	[138]
3	Soft carbon||graphite	Insertion	1 m NaPF_6_ in EC/DMC (6:4, v/v)	54 at 1000 mA g^−1^ after 800 cycles	40 at 2000 mA g	[22a]
4	Phosphorus‐doped soft carbon||graphite	Insertion	1 m NaPF_6_ in EC/DMC (6:4, v/v)	81 at 1000 mA g^−1^ after 900 cycles	73 at 3000 mA g	[22b]
5	Hard carbon||graphite	Insertion	2.55 m NaTFSI in TMP	34.5 at 500 mA g^−1^ after 200 cycles (cathode)	/	[90]
6	Carbon molecular sieve||KS_6_ graphite	Insertion	1 m NaPF_6_ in EC/EMC (1:2, v/v)	≈150 at 500 mA g^−1^ after 500 cycles	≈110 at 2000 mA g	[139]
7	TiO_2_||graphite	Insertion	1 m NaPF_6_ in EC/EMC (1:2, v/v)	98 at 500 mA g^−1^ after 1400 cycles	102 at 1500 mA g	[93]
8	Na_2_Ti_3_O_7_@G||coronene	Insertion	1 m NaPF_6_ in EC/DEC (1:1, v/v)	80 at 500 mA g^−1^ after 5000 cycles	60 at 1000 mA g	[95]
9	FePO_4_||graphite	Insertion	1 m NaPF_6_ in PC/EMC (3:7, v/v)	111.8 at 0.2 A g^−1^ after 250 cycles	/	[96]
10	Sn||graphite	Alloying	1 m NaPF_6_ in EC/DMC/EMC (1:1;1, v/v/v)	70 at 200 mA g^−1^ after 400 cycles (cathode)	61 at 500 mA g	[18a]
11	Sn||EG	Alloying	1 m NaPF_6_ in EC/DMC/EMC (1:1;1, v/v/v)	≈94 at 500 mA g^−1^ after 600 cycles (cathode)	91.6 at 1200 mA g	[18b]
12	P@C||graphite	Alloying	1 m NaPF_6_ in EC/DMC/EMC (1:1;1, v/v/v) with 10% FEC	201.5 at 0.25 A g^−1^ after 140 cycles	120.6 at of 1000 mA g	[98]
13	MoS_2_@C||graphite	Conversion	1 m NaPF_6_ in EC/DMC/EMC (1:1;1, v/v/v)	55 at 200 mA g^−1^ after 200 cycles (cathode)	35 at 1000 mA g	[99]
14	MoS_2_@C||EG	Conversion	1 m NaPF_6_ in EC/DMC/EMC (1:1;1, v/v/v)	60 at 100 mA g^−1^ after 300 cycles (cathode)	38.5 at 1000 mA g	[101]
15	MoS_2_@C||EG	Conversion	1 m NaPF_6_ in EC/DMC/EMC (1:1;1, v/v/v)	40 at 1 A g^−1^ after 500 cycles (cathode)	45 at 2000 mA g	[100]
16	MoS_2_@C||graphite	Conversion	1 m NaPF_6_ in EC/DMC (6:4 v v^−1^)	90.5 at 500 mA g^−1^ after 500 cycles	63.6 at 2000 mA g	[140]
17	SnP_2_O_7_||KS_6_ graphite	Conversion	1 m NaPF_6_ in EC/DMC/EMC (4:3;2, v/v/v)	≈70 at 2 A g^−1^ after 1000 cycles (cathode)	65 at 3000 mA g	[102]

a) EC: ethylene carbonate; EMC: ethyl‐methyl carbonate; PC: propylene carbonate; DMC: dimethyl carbonate; NaTFSI: sodium bis (trifluoromethanesulfonate) imide; TMP: trimethyl phosphate; DEC: diethyl carbonate.

### 4.1 Insertion Anodes for SDIBs

#### 4.1.1 Carbonaceous Materials

The anode materials with excellent sodium storage capacity in the low voltage range can match the cathode materials well, leading to a high voltage platform and energy density of SDIBs.^[^ ^18^, ^88^ ^]^ Thus, carbonaceous material with good sodium storage properties is a promising anode material for SDIBs. Wang et al. first reported the hard carbon derived from pine needles (PNC) as anode for SDIBs.^[^ ^89^ ^]^ The working mechanism of this SDIB can be demonstrated as follows: during the charging process, Na^+^ cations in the electrolytes intercalate into the PNC anode, whereas PF_6_^−^ anions intercalate into the graphite cathode; during the discharging process, both Na^+^ and PF_6_^−^ leave the anode and cathode, respectively, and come back into the electrolytes. The PNC exhibited stable cycling performance, with a capacity of 127 mAh g^−1^ at 500 mA g^−1^ after 1000 cycles. Due to the high voltage platform of 4 V, this SDIB could deliver a high energy density of 200 Wh kg^−1^, which is comparable with LIBs. Later, Cao and coworkers also fabricated the SDIBs with hard carbon as anode and graphite as cathode.^[^ ^90^ ^]^ Their research indicated that by using the NaTFSI salt dissolved in trimethyl phosphate (TMP) solvent as electrolytes, a TFSI‐derived inorganic SEI film could form on the surface of hard carbon, which can prevent the further reductive degradation of TMP and improve the Coulombic efficiency.

**Figure 8:**
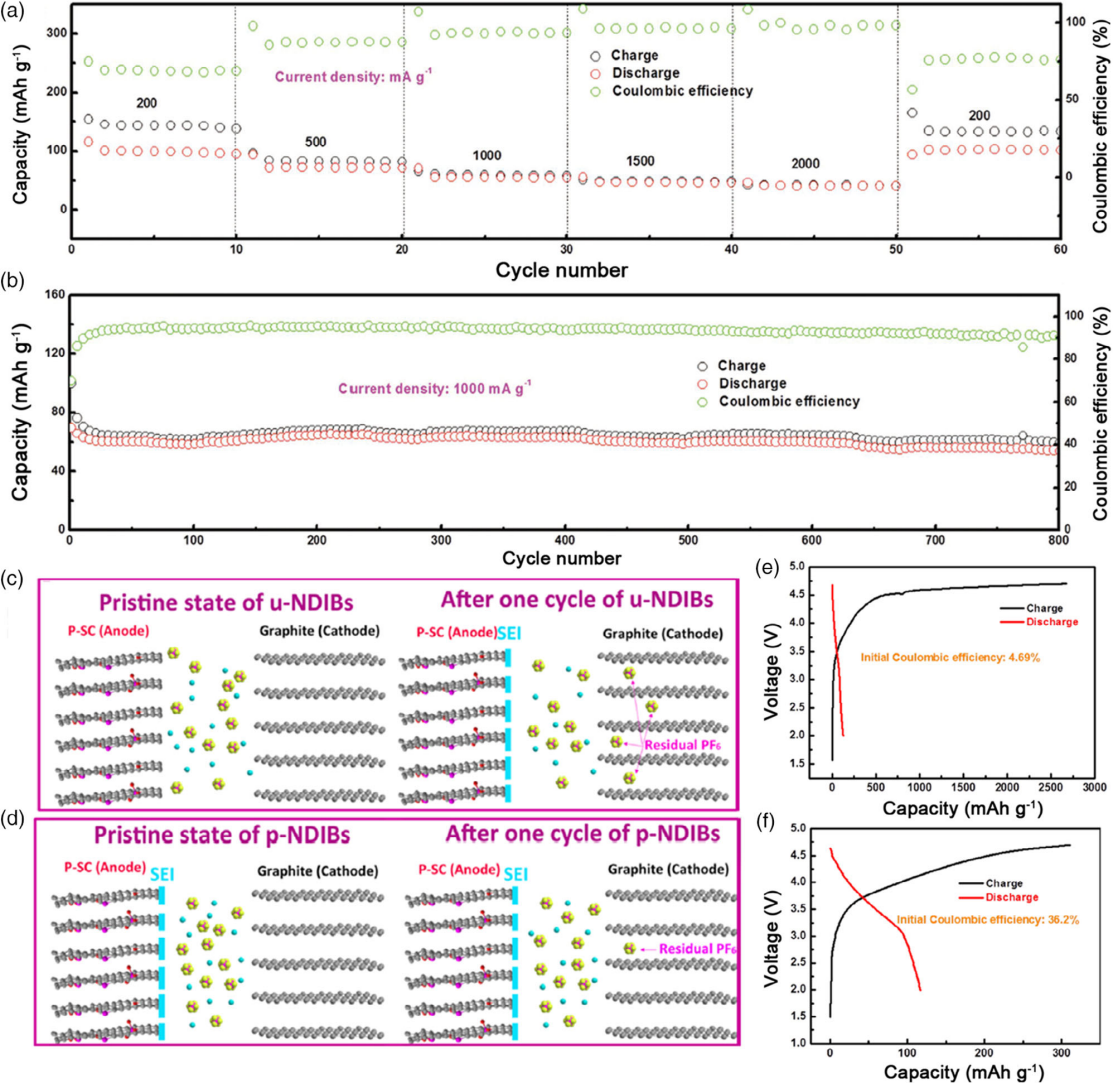
a) Rate capability and b) long cycling performance of the SDIBs based on soft carbon anodes with the voltage cut‐off from 2.0 to 4.7 V. a,b) Reproduced with permission.^[22a]^ Copyright 2017, Wiley-VCH. Schematic illustration of the working mechanism of c) SDIBs without presodiation process (denoted as u‐NDIBs) and d) SDIBs with presodiation process (denoted as p‐NDIBs) after one cycle, respectively. Initial charge/discharge profile of e) u‐NDIBs and f) p‐NDIBs at 500 mA g^−1^. c–f) Reproduced with permission.^[22b]^ Copyright 2018, American Chemical Society.

Compared with hard carbon, soft carbon has better electrical conductivity, leading to superior rate capability of the batteries.^[^ ^46^ ^]^ Lu and coworkers first reported the fabrication of SDIBs with soft carbon as anodes.^[22a]^ Coupled with graphite cathode, this SDIB delivered a high capacity of 103 mAh g^−1^ at 200 mA g^−1^, 40 mAh g^−1^ at 2000 mA g^−1^, and stable cycling performance for over 800 cycles (**Figure** **8**a,b). However, a large amount of Na^+^ would be consumed during the formation of SEI, leading to extremely low ICE of this SDIB. To solve this problem, they developed a phosphorus‐doped soft carbon (P‐SC) anode combined with a presodiation process in the following works.^[22b]^ For the SDIBs without a presodiation process, a large amount of Na^+^ were consumed to form the SEI in the initial cycles, leading to numerous PF_6_^−^ to be stranded in the graphite cathode (Figure 8c). However, by applying the presodiation strategy, SEI was first carried out on the anode (cycling for one cycle in half cells), which could reduce the Na^+^ consumption as well as retention of PF_6_^−^ in the graphite (Figure 8d), thus leading to a relatively high ICE. In addition, the P atom doping can further improve the electrical conductivity of the material and enhance the Na^+^ storage property. As a result, the presodiated P‐SC showed an enhanced ICE of about 36.2%, which was far more superior than the unsodiated P‐SC, about 4.69% (Figure 8e,f). This SDIB also displayed excellent cycling performance, with a capacity of 81 mAh g^−1^ at 1000 mA g^−1^ after 900 cycles corresponding to 81.8% of the initial capacity retention. Their research indicated that the offset initial sodium loss might be an effective way to improve the ICE of SDIBs.

#### 4.1.2 Other Insertion Anodes for SDIBs

TiO_2_ shows good application prospects as an intercalation anode for SIBs due to its low cost and stable structure. The sodiation plateau of TiO_2_ is about 0.8 V versus Na/Na^+^, which can circumvent the growth of sodium dendrite, thus leading to high safety performance of the batteries.^[^ ^91^ ^]^ In 2010, Yoshio and coworkers pioneered the work of studying the electrochemical performance of TiO_2_ in lithium dual‐ion batteries (LDIBs), which can retain 80% of the initial capacity after 30 cycles.^[^ ^92^ ^]^ Recently, Wang et al. constructed the SDIBs with anatase TiO_2_ as anode and graphite as cathode.^[^ ^93^ ^]^ Their research showed that PF_6_^−^ inserted into graphite obviously faster than Na^+^ inserted into TiO_2_, leading to the mismatching between anode and cathode. Therefore, modifying the morphology and structure of TiO_2_ or electrolytes composition to improve the Na^+^ diffusion rate may be an effective strategy to further enhance the electrochemical performance of TiO_2_‐graphite SDIBs. In addition, Na_2_Ti_3_O_7_ has been demonstrated as potential intercalation anodes for SIBs.^[^ ^94^ ^]^ Based on the advantages of Na_2_Ti_3_O_7_ in Na^+^ storage, Ji and coworkers fabricated the SDIBs using reduced‐graphene‐oxide‐modified Na_2_Ti_3_O_7_ (NTO@G) as anode and coronene (C_24_H_12_) as cathode.^[^ ^95^ ^]^ During the charging process, Na^+^ and PF_6_^−^ in the electrolytes would intercalate into anode and cathode, respectively, whereas the aforementioned process is reversed during the discharging process. The electrochemical reaction mechanism of NTO@G anode and C_24_H_12_ cathode are in accordance with the intercalation reactions as shown

(7) $$\text{Anode}:\;{\text{Na}}_{2}{\text{Ti}}_{3}O_{7}+x{\text{Na}}^{+}+xe^{-}↔{\text{Na}}_{2+x}{\text{Ti}}_{3}O_{7}$$

(8) $$\text{Cathode}:\;yC_{24}H_{12}+x{{\text{PF}}_{6}}^{-}↔yC_{24}H_{12}{\left({\text{PF}}_{6}\right)}_{x}+xe^{-}$$

(9) $$\text{Overall}:\;yC_{24}H_{12}+{\text{Na}}_{2}{\text{Ti}}_{3}O_{7}+x{\text{Na}}^{+}+x{{\text{PF}}_{6}}^{-}↔yC_{24}H_{12}{\left({\text{PF}}_{6}\right)}_{x}+{\text{Na}}_{2+x}{\text{Ti}}_{3}O_{7}$$

In the voltage window of 1.5–3.5 V, this SDIB exhibited a capacity of 160 mAh g^−1^ at 50 mA g^−1^ and ultralong cycling life for over 5000 cycles.

As discussed in the previous section, due to the irreversible electrolyte consumption by forming SEI on the anode, the ICEs of SDIBs are usually very low. Although the presodiation strategy can improve the ICE, it would increase the steps and cost of assembling batteries. Li et al. found that using FePO_4_ as anode for SDIBs will not cause the reductions of the electrolytes.^[^ ^96^ ^]^ Due to the high voltage platform of FePO_4_, the electrolytes would not be restored before the cut‐off voltage, leading to no SEI formation on the anode. As a result, this FePO_4_‐graphite SDIBs displayed ultrahigh ICE of 99.8%. In addition, the SDIBs delivered a stable cycling performance, with a capacity of 110 mAh g^−1^ at 0.2 A g^−1^ after 250 cycles.

### 4.2 Alloying Anodes for SDIBs

Sn has been intensively investigated as one of the promising alloying anode for SIBs. Based on the advantages of Sn in SIBs, our group first investigated the electrochemical performance of Sn as anode for SDIBs using Sn foil as the anode, expanded graphite (EG) as the cathode.^[18a]^ The working mechanism of this SDIB can be summarized as follows

(10) $$\text{Anode: Sn}+{\text{Na}}^{+}+e^{-}↔\text{NaSn}$$

(11) $$\text{Cathode}:\;xC+{{\text{PF}}_{6}}^{-}↔C_{x}({\text{PF}}_{6})+e^{-}$$

(12) $$\text{Full cell reaction}:\;\text{Sn}+xC+{\text{Na}}^{+}+{{\text{PF}}_{6}}^{-}↔\text{NaSn}+C_{x}({\text{PF}}_{6})$$

**Figure 9:**
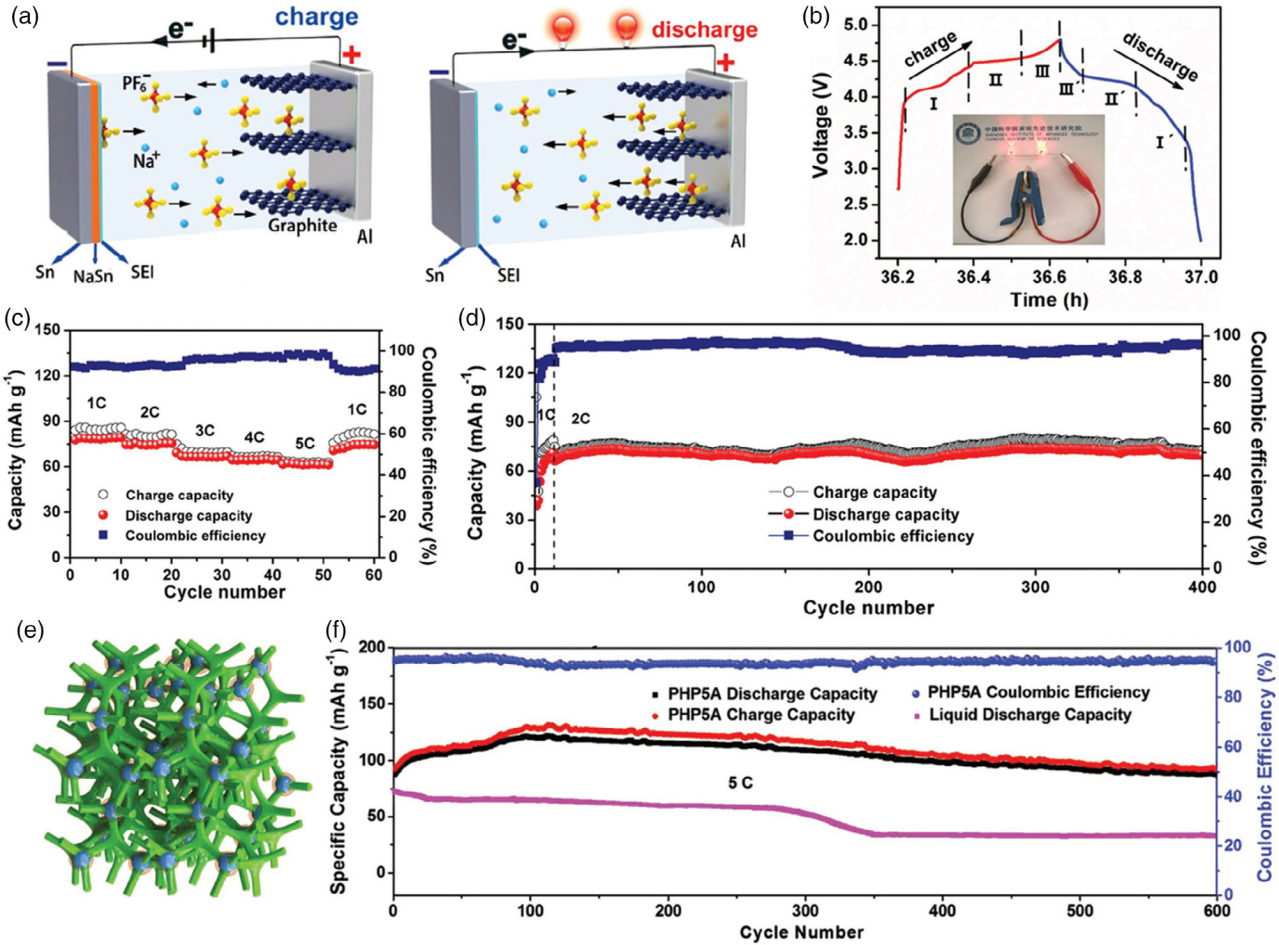
a) Schematic illustration of the configuration and working mechanism of Sn‐EG SDIBs. b) Galvanostatic charge–discharge curves of Sn‐EG SDIBs. Inset is a photograph showing that the Sn‐EG SDIBs could light up two red LEDs in series. c) Rate capability, and d) cycling performance of Sn‐EG SDIBs. a–d) Reproduced with permission.^[18a]^ Copyright 2016, Wiley-VCH. e) Schematic illustration of the 3D porous quasi‐solid‐state polymer electrolytes. f) Comparison of cycling performance between SDIBs with quasi‐solid‐state electrolytes and conventional liquid electrolytes. e,f) Reproduced with permission.^[18b]^ Copyright 2019, Wiley-VCH.

During the charging process, Na^+^ move to the anode and alloy with Sn to form Na–Sn alloy, whereas PF_6_^−^ move to cathode and intercalate into the graphite layers to form graphite intercalation compounds (GICs); during the discharge process, the reversed reaction happens (**Figure** **9**a). Due to the cation–anion dual‐ion strategy, this SDIB delivered a high working voltage of about 4.1 V, which could enable the battery to light up two red light‐emitting diodes (LEDs) in series (Figure 9b). The SDIBs delivered superior rate capability of 61 mAh g^−1^ at 5 C (calculated based on EG) and stable capacity retentions of 94% for 400 cycles (Figure 9c,d). In addition, we also reported a flexible quasi‐state SDIB constituted of Sn metal anode, EG cathode, and quasi‐solid‐state electrolytes.^[18b]^ Due to the 3D porous structure of the quasi‐solid‐state electrolytes (Figure 9e), this SDIB shows a high ionic conductivity of about 1.3 × 10^−3^ S cm^−1^, leading to fast ionic migration of cations and anions. In addition, the 3D porous network quasi‐solid‐state electrolyte is also in favor of the uniform distribution of electrochemical stress on Sn anode during the cycling process. As a result, this quasi‐state SDIB delivered a high capacity of 96.8 mAh g^−1^ at 5 C (calculated based on EG), as well as outstanding cycling stability for over 600 cycles (Figure 9f). Although Sn foil displays good electrochemical performance as anode for SDIBs, it is worth noting that to ensure a long cycling lifespan, the capacity of Sn is obviously excessive in case of the anode capacity attenuation caused by the volumetric expansion. However, this will result in the waste of anode material as well as lowering the energy density of SDIBs. Fabricating Sn–carbon composite with nanostructure and matching the capacity between anode and cathode may be an effective strategy to solve this problem and further promote the energy density of Sn anode‐based SDIBs.

As another typical alloying material, RP shows good potential application prospect in sodium storage due to its high theoretical specific capacity (2596 mAh g^−1^), appropriate electrochemical plateaus (0.45 V vs Na/Na^+^), and abundant reserves in the Earth's crust. However, RP exhibits a low electronic conductivity of about 10^−14^ S cm^−1^ and suffers from huge volumetric change during the cycling process, leading to fast capacity decay.^[^ ^97^ ^]^ Combining the highly conductive carbon matrix with small RP particles can effectively solve these problems and improve the storing capacity of sodium, which has achieved considerable progress in SIBs. Recently, Wang and coworkers constructed a novel SDIB using RP/CNT@RGO composite as anode and graphite as cathode.^[^ ^98^ ^]^ Through the ex situ XRD, HRTEM, and X‐ray photoelectron spectroscopy techniques, the author demonstrated that the electrochemical mechanism between Na^+^ and RP anode via a reversible Na–P alloying reaction. This SDIB exhibited a reversible specific capacity of 373 mAh g^−1^ at 0.1 A g^−1^ (calculated based on RP). In general, the investigation of RP as anode for SDIBs is just beginning, and there is still a lot of room for electrochemical performance improvement. Therefore, innovations in materials architecture design and further mechanism research are needed to realize phosphorus‐based SDIBs with outstanding electrochemical properties.

### 4.3 Conversion Anodes for SDIBs

**Figure 10:**
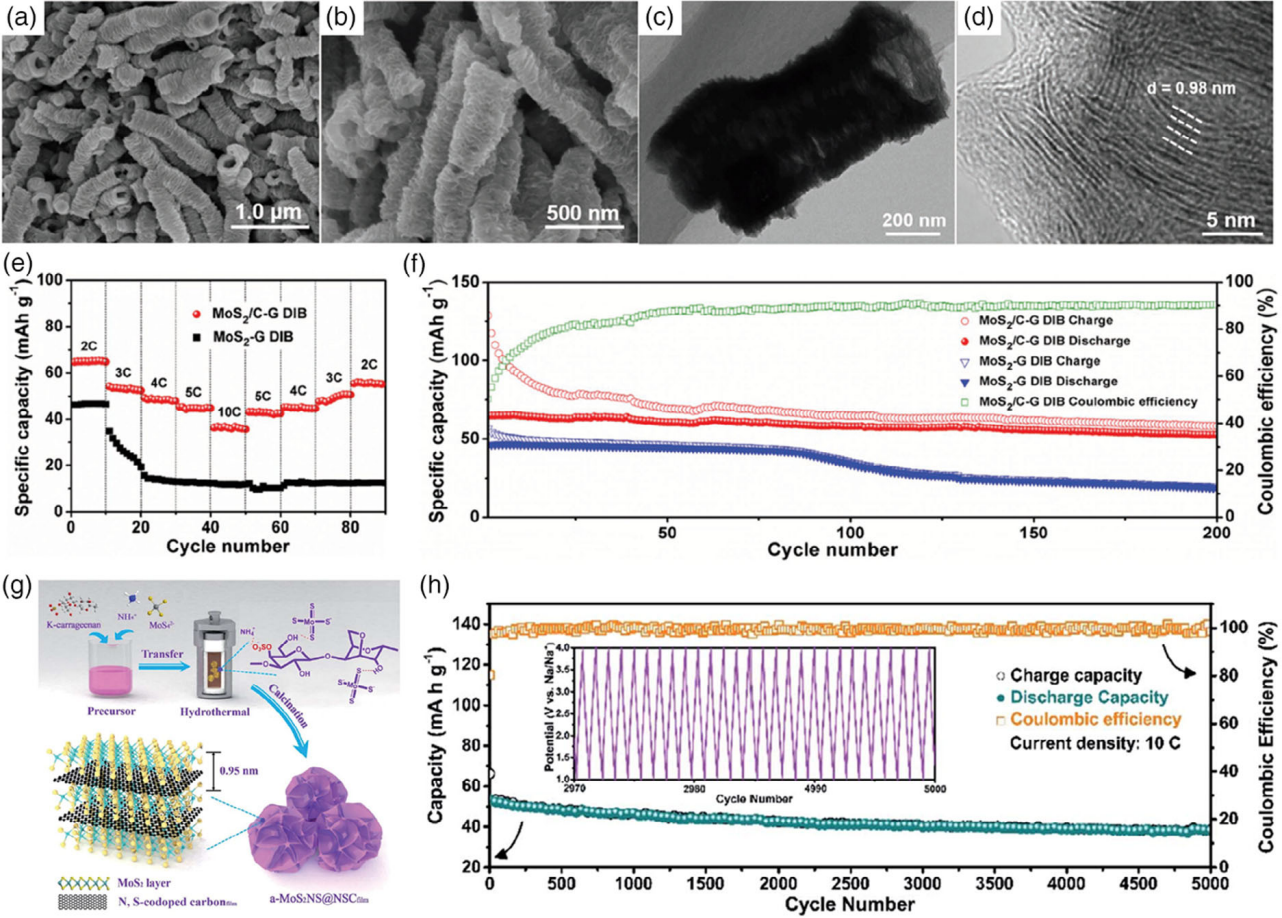
a,b) SEM images of the MoS_2_/C nanotube with different magnifications. c,d) TEM and HRTEM images of the MoS_2_/C nanotube. e) Comparison of rate capability of MoS_2_/C nanotube and pure MoS_2_ as anode for SDIBs. f) Comparison of cycling performance of MoS_2_/C nanotube and pure MoS_2_ as anode for SDIBs. a–f) Reproduced with permission.^[^ ^99^ ^]^ Copyright 2018, Wiley-VCH. g) Schematic illustration of the preparation procedure of a‐MoS_2_NS@NSC_film_ nanohybrids. h) Cycling performance of SDIBs with a‐MoS_2_NS@NSC_film_ anode and EG cathode at 10 C for 5000 cycles. Inset is the galvanostatic charge/discharge curves between 4970 and 5000 cycles. g,h) Reproduced with permission.^[^ ^101^ ^]^ Copyright 2019, Royal Society of Chemistry.

MoS_2_ has been widely investigated as the anode for LIBs and SIBs. In recent years, researchers tried to use MoS_2_ as anode for SDIBs and achieved remarkable results. Using the MoS_2_/C nanocomposite as anode and EG as cathode, our group first reported the MoS_2_‐EG SDIBs.^[^ ^99^ ^]^ The prepared MoS_2_/C nanocomposite has a hierarchical penne‐like nanotube structure with carbon evenly coating (**Figure** **10**a–c), leading to more reactive sites. In addition, this MoS_2_/C nanotube provided an enlarged (002) interlayer spacing about 0.98 nm of 2 H‐MoS_2_ (Figure 10d), which could improve the Na^+^ diffusion kinetics. In the voltage range of 1.0–4.0 V, this SDIB exhibited good rate capability of 45 mAh g^−1^ at 5 C and stable cycling performance for 200 cycles (Figure 10e,f). Recently, Chu and coworkers designed the hierarchical hollow spheres assembled from few‐layer MoS_2_ nanosheets with N‐doped carbon coating (MoS_2_@NC HHSs) and investigated its electrochemical performance as anode for SDIBs.^[^ ^100^ ^]^ The hollow spherical structure and N‐doped carbon‐coating layer can improve the reaction kinetics of SDIBs and ensure stable mechanical properties during the sodiation process. As a result, this SDIB delivered stable cycle retention for 500 cycles. Similar to this work, Wen and coworkers reported the fabrication of coral‐like nanohybrids consisting of layer‐by‐layer stacked MoS_2_ nanosheets with N and S codoped carbon coating film (a‐MoS_2_NS@NSC_film_) by a solvothermal method, as shown in Figure 10g.^[^ ^101^ ^]^ Due to the carbon film modification, the MoS_2_ nanosheets exhibited an expanded layer space of 0.95 nm. In addition, the codoping of N and S heteroatoms can significantly enhance the electrical conductivity of the composite. Combining these effects, the SDIBs with a‐MoS_2_NS@NSC_film_ anode and EG cathode displayed ultralong cycling stability for 5000 cycles (Figure 10h). All these results indicate that MoS_2_ shows good potential application feasibility in SDIBs; however, challenges such as relatively low specific capacity and storage mechanisms remain to be addressed to further improve the energy density and power density of SDIBs.

**Figure 11:**
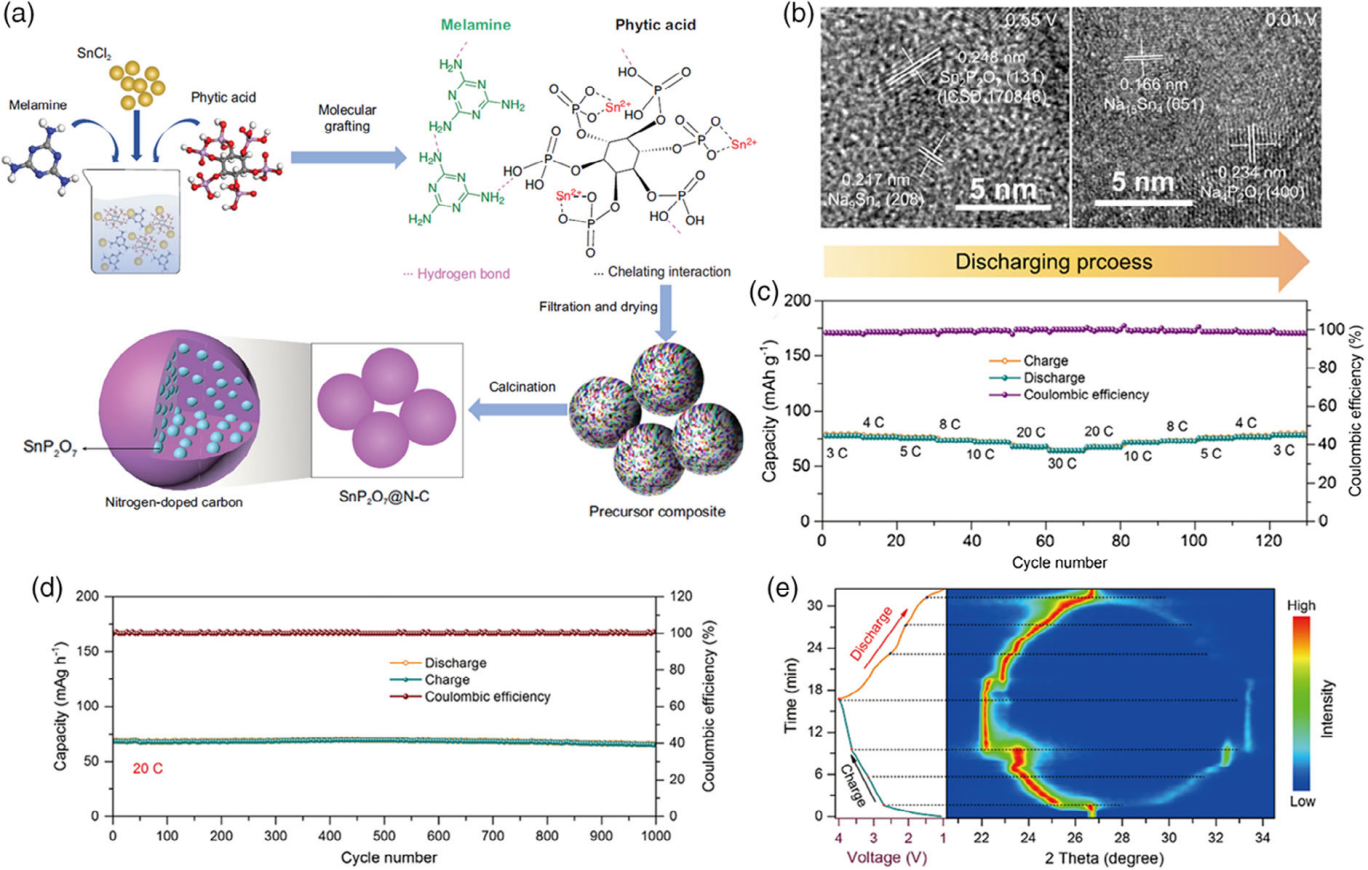
a) Schematic illustration of the preparation procedure of SnP_2_O_7_@N‐C composite. b) HRTEM images of SnP_2_O_7_@N‐C at discharging states of 0.55 and 0.01 V, respectively. c) Rate capability of SDIBs with SnP_2_O_7_@N‐C anode and graphite cathode at various current rates from 3 to 30 C. d) Cycling performance of the SDIBs at 20 C for 1000 cycles. e) Galvanostatic charge–discharge curve of the SDIBs at 3 C and the corresponding in situ XRD contour of graphite. a–e) Reproduced with permission.^[^ ^102^ ^]^ Copyright 2020, Oxford University Press.

As a typical alloying type material, Sn exhibits high specific capacity, but huge volumetric change during the sodiation process.^[^ ^56^, ^58^ ^]^ Numerous Sn‐based nanostructures, carbon composites, and compounds have been synthesized to solve this problem and remarkable results have been achieved in the SIBs system.^[^ ^53^, ^55^, ^56^, ^57^, ^58^ ^]^ However, only Sn foil has been investigated in SDIBs system, which is far from satisfactory.^[18a]^ Recently, our group prepared a Sn‐based compound (SnP_2_O_7_) and investigated its electrochemical performance as anode for SDIBs.^[^ ^102^ ^]^ In this composite, SnP_2_O_7_ nanodots were in situ implanted in an N‐doped carbon matrix (SnP_2_O_7_@N‐C) through a molecular grafting strategy, as shown in **Figure** **11**a. According to the thermogravimetric analysis, the prepared SnP_2_O_7_@N‐C showed a low carbon content of about 4.4 wt%. It should be noted that although the carbon matrix could enhance electrical conductivity and provide a buffer for the volumetric variation of the active material, superfluous carbon would reduce the content of the active material, leading to a low specific capacity of the electrodes. Therefore, the content of active material should be increased as much as possible under the premise of ensuring the electrical conductivity and stability of the electrode. We also found that SnP_2_O_7_ can react with Na^+^ to form Na_4_P_2_O_7_ and Na_15_Sn_4_ during the discharging process, as shown in the HRTEM images of the anode materials at different discharging states (Figure 11b), indicating a conversion reaction mechanism of SnP_2_O_7_ during the sodiation process. The SnP_2_O_7_@N‐C anode showed outstanding electrochemical performance in SDIBs pairing with graphite cathode, which exhibited high rate capability up to 30 C, as well as a long‐term cycling lifespan at 20 C for over 1000 cycles (Figure 11c,d). In addition, the insertion/extraction behavior of PF_6_^−^ in cathode was also proved through the in situ XRD technique (Figure 11e), indicating good compatibility between SnP_2_O_7_@N‐C anode and graphite cathode.

## 5 Conclusions

Due to the increasing energy demands and serious environmental problems, researchers have devoted massive efforts to developing clean and sustainable energy technology. Because of the natural abundance and low cost of sodium resources, Na^+^ cells, including SIBs and SDIBs have received more and more attention as promising energy storage devices in the future. However, the sluggish reaction kinetics of Na^+^, structural instability of Na^+^ host electrode materials, as well as the low energy density significantly hinder the development of advanced Na^+^ cells. Therefore, the development of novel attractive electrode materials, especially anode materials, is important and in urgent demand. In this Review, we have discussed the most recent progress on anode materials for high‐performance SIBs, including material preparation methods, structural properties, reaction mechanisms, and electrochemical properties. Among the insertion anodes, hard carbon exhibits superior Na^+^ storage capability due to its enlarged interlayer spacing. The main issue of hard carbon is the poor rate capability, which may be solved by the strategies of heteroatom doping. In addition, because of the insertion reaction mechanism, hard carbon has a relatively low sodium storage capacity, leading to inferior energy density of the batteries. This makes hard carbon unsuitable for the applications requiring high energy density, such as portable electronics and electric vehicles. But due to its low cost, mature production chain, and stable cycling performance, hard carbon still has huge application prospects in the field requiring low cost and long cycling lifespan rather than high energy density, such as large‐scale stationary energy storage. Compared with insertion anodes for SIBs, the alloying and conversion anodes can exhibit much higher specific capacity. For these materials, the huge volumetric expansion during the sodiation process is the main obstacle to its high performance, which could cause a rapid capacity decay of the SIBs. As discussed in this Review, compositing with carbon material, optimizing the structure design of electrode materials, as well as choosing proper electrolytes could be effective strategies to improve this problem.

Furthermore, we also summarized the recent progress on anode materials in the SDIB system for the first time. Compared with SIBs, SDIBs are cheaper and more environmentally friendly due to their ability to directly use carbonaceous materials as the cathode. As SDIBs are currently at a quite primary stage, only a few materials (including hard carbon, soft carbon, TiO_2_, Na_2_Ti_3_O_7_, FePO_4_, Sn, P, MoS_2_, and SnP_2_O_7_) have been investigated as the anode for SDIBs. In principle, the materials that can store Na^+^ in SIBs could also be used as the anode for SDIBs, which makes it possible to apply the successful experience of exploiting anode materials on SIBs systems to the SDIBs systems. However, compared with the “rocking chair” SIBs, SDIBs have a different working mechanism, in which both Na^+^ and anions are stored in anode and cathode materials, respectively. Thus, it must be considered that the rate of reaction between the Na^+^ and the anode needs to match the rate of reaction between the anion and the cathode.

Under the unremitting efforts of scientific researchers, significant progress has been made in the development of SIBs and SDIBs for the past few years. However, there are still several challenges that need to be tackled before taking a step toward large‐scale commercial application. Up to now, most of the researches on anode materials with excellent performance are based on nanomaterial technology. It is true that compared with bulk materials, nanomaterials can obtain better electrochemical performance such as high reversible capacity, superior rate capability, and stable cycling performance. Nevertheless, little attention has been paid to the shortcomings of nanomaterials. For instance, due to the high specific surface area of nanomaterials, a large number of SEI would form on the surface of the nanoscale anodes, thus leading to largely irreversible capacity loss during the initial cycles. This would cause a low ICE of the SIBs, which is adverse to the commercialization of SIBs. Therefore, more studies on rational structure design, material surface modification, as well as electrolyte optimization are needed to improve the ICE of anodes based on nanomaterials. In addition, due to the additional space to accommodate the volume deformation in the material matrix during the sodiation process, the nanomaterials with porous, hollow, or yolk–shell structures would cause a low tap density of the electrode, which would significantly reduce the volumetric capacity of the batteries. To solve this problem, constructing the electrode with a freestanding structure may be an effective approach, for which can not only increase the load per unit volume of active material but also further reduce the cost of cells as binder and current collector are not used.

As for anodes in SDIBs, only a few materials have been investigated up to now; thus, the exploration of appropriate anode materials with outstanding electrochemical performance is in urgent need. To further enrich the anode materials for SDIBs, it is important to understand the reaction kinetics between the Na^+^ in anode and anions in the cathode, as well as the working mechanism of SDIBs. Combining theoretical calculations with experimental characterizations could be a useful way to guide the structure design, electrochemical performance optimization, and prediction of new anode materials. Furthermore, some advanced in situ characteristic techniques, such as XRD, TEM, Raman spectroscopy, and neutron diffraction, could also be used to investigate the changes in crystalline structure and kinetics of electrode materials during the charging/discharging process, which could provide new insights for in‐depth understanding the reaction mechanisms of electrodes and developing novel anode materials.

## Conflict of Interest

The authors declare no conflict of interest.
